# Cytoskeletal disarray increases arrhythmogenic vulnerability during sympathetic stimulation in a model of hypertrophic cardiomyopathy

**DOI:** 10.1038/s41598-023-38296-2

**Published:** 2023-07-12

**Authors:** Henrietta Cserne Szappanos, Helena M. Viola, Danica W. Ito, Seakcheng Lim, Melissa Mangala, Mira Holliday, Samantha Barratt Ross, Christopher Semsarian, Adam Hill, Rose E. Dixon, Livia C. Hool

**Affiliations:** 1 School of Human Sciences, University of Western Australia, Crawley, WA Australia; 2 Department of Physiology and Membrane Biology, University of California, Davis, CA USA; 3 Agnes Ginges Centre for Molecular Cardiology, Centenary Institute, Sydney, Australia; 4 Sydney Medical School, University of Sydney, Sydney, Australia; 5 Victor Chang Cardiac Research Institute, Sydney, NSW Australia; 6 Department of Cardiology, Royal Prince Alfred Hospital, Sydney, Australia

**Keywords:** Biophysics, Cell biology, Genetics, Physiology, Stem cells, Cardiology, Diseases, Medical research, Pathogenesis

## Abstract

Familial hypertrophic cardiomyopathy (FHC) patients are advised to avoid strenuous exercise due to increased risk of arrhythmias. Mice expressing the human FHC-causing mutation R403Q in the myosin heavy chain gene (*MYH6*) recapitulate the human phenotype, including cytoskeletal disarray and increased arrhythmia susceptibility. Following in vivo administration of isoproterenol, mutant mice exhibited tachyarrhythmias, poor recovery and fatigue. Arrhythmias were attenuated with the β-blocker atenolol and protein kinase A inhibitor PKI. Mutant cardiac myocytes had significantly prolonged action potentials and triggered automaticity due to reduced repolarization reserve and connexin 43 expression. Isoproterenol *shortened* cycle length, and escalated electrical instability. Surprisingly isoproterenol did not increase Ca_V_1.2 current. We found alterations in Ca_V_1.2-β1 adrenergic receptor colocalization assessed using super-resolution nanoscopy, and increased Ca_V_1.2 phosphorylation in mutant hearts. Our results reveal for the first time that altered ion channel expression, co-localization and β-adrenergic receptor signaling associated with myocyte disarray contribute to electrical instability in the R403Q mutant heart.

## Introduction

Arrhythmias and premature sudden death are tragic sequelae in patients with inherited heart disease that can occur with increased sympathetic activity^1–3^. Familial hypertrophic cardiomyopathy (FHC) is a primary disorder of the myocardium characterized by cardiac hypertrophy in the absence of other loading conditions. It is an autosomal dominant condition caused by defects in many sarcomere protein encoding genes. The majority of disease-causing variants are located in beta myosin heavy chain (*MYH7*) and myosin binding protein C (*MYBPC3*)^4^. It is well recognized that the progression of FHC involves altered energy metabolism, myocyte remodeling, disorganization of cytoskeletal proteins and fibrosis, and results in major adverse cardiac events such as heart failure and sudden cardiac death^5–7^. Intense exercise is thought to promote ventricular tachyarrhythmias, therefore FHC patients are advised to avoid intense physical activity and competitive sport^8^. Although fibrosis and hypertrophy are recognized substrates for arrhythmias, alterations in the electrical properties of the cardiac myocyte and its response to adrenergic stimulation can also contribute to the genesis of ventricular arrhythmias and sudden cardiac death^1,9^.

In humans, the R403Q variant in *MYH7* causes a severe form of FHC characterized by early-onset and progressive myocardial dysfunction with a high incidence of sudden cardiac death^10^. Mice expressing the R403Q mutation in *MYH6*, encode the predominant myosin isoform in the adult mouse heart that is highly homologous in sequence with *MYH7*, develop hallmark features of hypertrophic cardiomyopathy from 30 weeks of age^5^. Homozygous mice are viable at birth and look anatomically normal, but die by day 7 with severe dilated cardiomyopathy. Mice heterozygous for the R403Q *MYH6* mutation (αMHC^403/+^) have a normal lifespan, and preserved cardiac function. Young heterozygous mice demonstrate myofibril disorientation, myocyte disarray^5^, alterations in L-type calcium channel kinetics and altered mitochondrial metabolic activity^7^ that precede the development of myocyte hypertrophy, myocyte injury and fibrosis. Regardless of the presence of hypertrophy, hearts exhibit impaired diastolic function, myocyte cytoskeletal disarray and altered energetics^11^.

Similar to FHC patients, αMHC^403/+^ hypertrophic mice can experience serious arrhythmias with vigorous exercise^5,12^. The pathophysiology contributing to the development of arrhythmias is unclear. A study using high-resolution optical mapping of αMHC^403/+^ hypertrophic hearts during ventricular pacing, found no direct correlation between the amount or the pattern of fibrosis and inducibility of arrhythmias^13^. Arrhythmia formation at the cellular level centers on two key concepts: altered calcium homeostasis and reduced repolarization reserve^14,15^. In addition to an increase in myofilament calcium sensitivity^16^, αMHC^403/+^ mice demonstrate a significant reduction in sarcoplasmic reticulum calcium content^7^ due to decreased expression of calsequestrin, triadin, junctin and ryanodine receptor 2 (RyR2), the proteins forming the cardiac calcium release unit^11^. Interestingly, although no differences in diastolic or systolic calcium concentrations were measured in cardiac myocytes isolated from pre-cardiomyopathic αMHC^403/+^ mice, calcium channel blockers such as diltiazem prevented development of the hypertrophy^11,17^. In addition an early remodeling of repolarizing K^+^ currents has been reported prior to the development of hypertrophy in the αMHC^403/+^ mouse that contributes to alterations in repolarization^18^. However the effect of sympathetic nervous system stimulation on arrhythmia formation is unclear.

The objective of this study was to investigate the mechanisms for induction of arrhythmias in the αMHC^403/+^ murine model of FHC with developed hypertrophy in the absence and presence of β-adrenergic receptor stimulation. We performed a suite of in vivo and in vitro studies and found that contrary to effects observed in wt hearts, action potentials in αMHC^403/+^ myocytes were prolonged and β-adrenergic receptor stimulation *shortened* the action potential while increasing the frequency of delayed afterdepolarizations and ventricular tachyarrhythmias. This was recapitulated in αMHC^403/+^ mice following *in* vivo challenge with isoproterenol. Consistent with the cytoskeletal disarray, Ca_V_1.2-β1 adrenergic receptor colocalization was altered assessed by super-resolution nanoscopy. Ca_V_1.2 was unresponsive to isoproterenol due to increased phosphorylation in mutant hearts and connexin 43 expression was significantly decreased. We conclude that altered ion channel expression, location and function contribute to altered β-adrenergic receptor signaling and increased automaticity. Our data demonstrate for the first time an association between cytoskeletal disarray and arrhythmia formation in the R403Q mutant heart.

## Results

### Sympathetic stimulation induces sustained arrhythmias in αMHC^403/+^ mice with a hypertrophic phenotype

**Figure 1:**
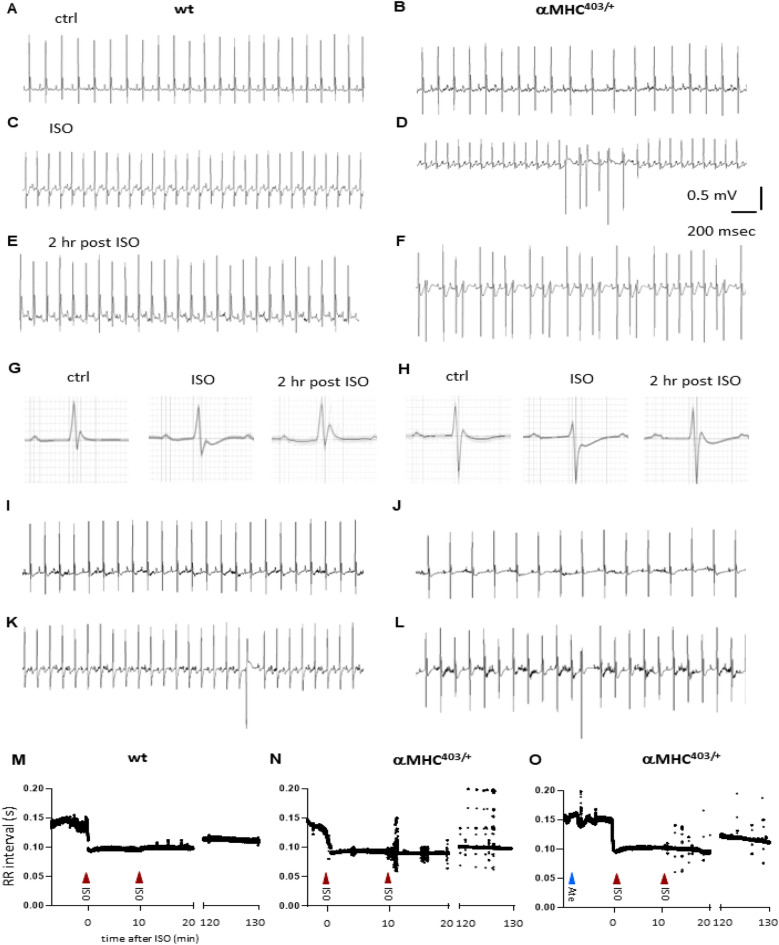
Representative electrocardiograms (ECG) before (**A**–**B**) during (**C**–**D**) and 2 h after (**E**–**F**) i. p. injection of 2 × 20 mg/kg ISO in a wt (**A**, **C**, **E**) and a αMHC^403/+^ mouse (**B**, **D**, **F**). Signal-averaged complexes show distinct effects of ISO on wt (**G**) and αMHC^403/+^ mouse (**H**). Representative ECG recordings in an αMHC^403/+^ mouse before (**I**) and after 1 mg/kg atenolol (**J**) and following ISO (**K**) treatment. (**L**) Recovery of the αMHC^403/+^ mouse 2 h post-ISO. (**M**–**O**) R–R intervals plotted against time for ISO treated wt (**M**), αMHC^403/+^ mouse (**N**) and αMHC^403/+^ mouse treated with atenolol (Ate) 10 min prior to ISO as indicated (**O**). The irregular RR intervals representing arrhythmias can be seen as dots following ISO.

First we examined the effect of the β-adrenergic receptor agonist isoproterenol (ISO) on arrhythmia inducibility in vivo. ECGs were recorded in 35–45 week old αMHC^403/+^ and wt mice before and after intraperitoneal injection of 20 mg/kg ISO. Isoproterenol has a half-life of 2.5 to 5 min^19^. To monitor arrhythmia inducibility for an extended time period, a second dose of 20 mg/kg ISO was applied 10 min after the first injection (Fig. 1A–D). The dose is well tolerated and does not induce myocardial damage^20^. Lead II ECGs were recorded using subdermal needle electrodes with no programmed electrical stimulation. Prior to administration of ISO, αMHC^403/+^ mice exhibited significantly longer QT, QT_c_ intervals and T_peak_ T_end_ duration compared to wt mice (Table 1). In the absence of ISO, single premature ventricular contractions (PVC) were occasionally recorded in αMHC^403/+^ mice. None of the mice exhibited atrial arrhythmias at baseline or following administration of isoproterenol.

**Table 1:** Electrocardiogram parameters recorded on wt and αMHC^403/+^ mice.

ECG parameters	wt						αMHC^403/+^					
	ctrl	ISO	2 h post-ISO	Ate	Ate + ISO	2 h post-Ate + ISO	ctrl	ISO	2 h post-ISO	Ate	Ate + ISO	2 h post-Ate + ISO
N	12	7	4	5	5	5	12	7	6	5	5	5
RR interval (ms)	111.8 ± 2.1	95.0 ± 1.1*	106.1 ± 4.4	142.6 ± 4.3*	94.9 ± 2.8*	109.3 ± 4.2	113.1 ± 2.8	93.8 ± 1.5^a^	97.5 ± 3.0^a^	167.4 ± 5.3^a^†	96.0 ± 2.5^a^	107.6 ± 3.6
heart rate (bpm)	543.8 ± 9.6	633.1 ± 6.8*	568.2 ± 23.8	423.5 ± 12.9*	634.7 ± 19.5*	551.4 ± 21.1	534.5 ± 13.0	640.4 ± 9.9^a^	618.0 ± 17.8^a^	361.2 ± 11.0^a^†	626.6 ± 16.1^a^	560.1 ± 18.9
PR interval (ms)	45.8 ± 1.7	38.2 ± 1.9*	41.6 ± 2.2	44.2 ± 1.5	40.5 ± 2.0	46.4 ± 3.2	46.7 ± 1.2	45.5 ± 1.7	41.1 ± 1.2	45.7 ± 1.4	42.8 ± 2.9	47.3 ± 2.6
P duration (ms)	12.3 ± 0.7	9.7 ± 0.6	12.4 ± 1.0	11.1 ± 0.8	10.3 ± 1.3	15.2 ± 2.5	11.6 ± 0.5	15.6 ± 3.5	9.6 ± 1.6	10.3 ± 0.7	9.0 ± 0.7	9.7 ± 0.9
QRS interval (ms)	8.8 ± 0.2	8.1 ± 0.3	9.5 ± 1.1	8.3 ± 0.5	9.4 ± 0.7	9.8 ± 0.5	7.7 ± 0.3	9.0 ± 0.6	8.1 ± 0.4	7.4 ± 0.2	7.8 ± 0.4	8.1 ± 0.4
QT interval (ms)	25.4 ± 1.1	25.9 ± 2.1	25.0 ± 5.7	49.8 ± 2.4*	32.6 ± 4.5	28.9 ± 3.7	52.4 ± 3.6†	44.0 ± 3.1†	53.0 ± 1.3†	54.7 ± 1.1	46.3 ± 1.7	52.0 ± 4.0†
QT_c_ interval (ms)	24.2 ± 0.9	26.6 ± 2.2	22.0 ± 3.8	42.1 ± 2.1*	33.5 ± 4.6	41.7 ± 6.0*^b^	49.3 ± 3.2†	45.6 ± 3.4†	53.8 ± 1.6†	45.5 ± 3.2	46.7 ± 1.7	50.7 ± 3.7
T_peak_T_end_ (ms)	12.9 ± 1.0	12.7 ± 1.9	9.4 ± 3.8	32.2 ± 4.8*	21.0 ± 4.2	21.4 ± 3.9	40.1 ± 3.6†	30.8 ± 3.4†	40.1 ± 1.4†	44.0 ± 1.2	32.8 ± 1.3	38.2 ± 4.0†
P ampl (mV)	0.11 ± 0.01	0.11 ± 0.01	0.11 ± 0.01	0.09 ± 0.01	0.10 ± 0.03	0.10 ± 0.01	0.09 ± 0.01	0.10 ± 0.02	0.08 ± 0.02	0.08 ± 0.01	0.08 ± 0.01	0.07 ± 0.01
R ampl (mV)	1.15 ± 0.02	0.99 ± 0.11	1.19 ± 0.09	1.06 ± 0.04	0.97 ± 0.05	1.07 ± 0.08	0.93 ± 0.07†	0.44 ± 0.07^a^†	0.57 ± 0.13^a^†	1.18 ± 0.08	0.66 ± 0.07	0.94 ± 0.08#
T ampl (mV)	0.26 ± 0.02	0.19 ± 0.10	0.37 ± 0.10	0.17 ± 0.03*	-0.09 ± 0.07*	0.19 ± 0.08	0.20 ± 0.02	0.04 ± 0.09^a^	-0.07 ± 0.13^a^†	0.14 ± 0.04	-0.09 ± 0.02^a^	0.03 ± 0.07^a^
relative occurrence of irregular beats (1/s)	0.0006 ± 0.0004	0.0048 ± 0.0037	0.0020 ± 0.0011	0.0001 ± 0.0001	0.0021 ± 0.0012	0.0018 ± 0.0014	0.0009 ± 0.0004	0.0165 ± 0.0056^a^	0.0225 ± 0.0077*†	0.0014 ± 0.0009	0.0059 ± 0.0036	0.0093 ± 0.0029

Data were acquired prior to and following in vivo treatment of 2 × 20 mg/kg isoproterenol (ISO) i.p. in the absence or the presence of 1 mg/kg atenolol (Ate) i.p. Values are means ± SEM. Brown-Forsyth and Welch ANOVA was used to analyze differences between wt and αMHC^403/+^ mice followed by a Dunn’s test to correct for multiple comparisons *p < 0.05 versus ctrl wt, ^a^*p* < 0.05 versus ctrl αMHC^403/+^, †*p* < 0.05 wt versus αMHC^403/+^ under the same condition, ^b^*p* < 0.05 versus 2 h post ISO wt, #*p* < 0.05 versus 2 h post ISO αMHC^403/+^.

Following ISO treatment, both wt and αMHC^403/+^ mice exhibited a significantly higher heart rate shown as an increase in beats per minute (Table 1). Although QT and QT_c_ intervals, and T_peak_ T_end_ duration were slightly shortened in αMHC^403/+^ mice following ISO, all remained significantly longer compared to wt mice, while R and T amplitudes became significantly reduced (Table 1, Fig. 1G,H). Arrhythmic events were calculated as number of irregular beats each second of recording and included single PVC’s and ventricular tachycardia (Fig. 1D). One of the eight αMHC^403/+^ mice suffered cardiac arrest shortly after the ISO injection and significant arrhythmias were recorded in six of the seven surviving αMHC^403/+^ mice (Table 1). One of the αMHC^403/+^ mice showed no spontaneous or inducible ventricular arrhythmias. Our data are consistent with reports of arrhythmias and sudden death in αMHC^403/+^ mice following vigorous swimming^5^.

A final ECG was taken two hours following administration of ISO. The relative occurrence of arrhythmic events in αMHC^403/+^ mice was tenfold higher 2 h post-ISO than wt mice challenged with ISO with a higher heart rate (Fig. 1E,F,N vs. M and Table 1). In addition we found that the 6 αMHC^403/+^ mice that exhibited prolonged exacerbated arrhythmic events demonstrated poor recovery and lethargy 2 h following ISO injection observed as difficulty mobilizing and decreased activity moving around the cage assessed using BAR (bright, alert, responsive) animal monitoring criteria and scoring (see original Monitoring Sheets in Supplementary Information File). This was in contrast to wt mice that remained active and spontaneously groomed and fed following ISO injection.

Consistent with the development of a hypertrophic phenotype, αMHC^403/+^ mice displayed a significant increase in left ventricular posterior wall thickness and significant decrease in left ventricular internal diameter compared to wt mice (Table 2 and Fig. S1A,B). Stroke volume and diastolic parameters were reduced in mutant mice consistent with previous reports^21^. In wt mice, ISO treatment induced a significantly greater decrease in left ventricular internal diameter at end systole (LVIDs) and end systolic volume (ESV) compared with mutant hearts. In αMHC^403/+^ mice treated with ISO the change in ejection fraction (14% increase) was less marked than wt (22%, Table 2). These findings confirm that the αMHC^403/+^ mouse heart has difficulty complying with the increased contractile demands imposed during sympathetic nervous system stimulation.

**Table 2:** Echocardiography parameters recorded on wt and αMHC^403/+^ mice.

ECHO parameters	wt				αMHC^403/+^			
	ctrl	ISO	Ate	Ate + ISO	ctrl	ISO	Ate	Ate + ISO
N	12	7	5	5	12	7	5	5
IVSd (mm)	0.77 ± 0.01	0.84 ± 0.01*	0.71 ± 0.03	0.80 ± 0.01	0.91 ± 0.02†	0.95 ± 0.02†	0.84 ± 0.01†	0.88 ± 0.02†
LVIDd (mm)	3.37 ± 0.06	3.16 ± 0.09	3.48 ± 0.13	2.93 ± 0.09*	2.95 ± 0.08†	2.55 ± 0.06^a^†	3.39 ± 0.16^a^	2.79 ± 0.14
LVPWd (mm)	0.80 ± 0.01	0.89 ± 0.03*	0.74 ± 0.03	0.84 ± 0.01	0.97 ± 0.03†	1.03 ± 0.03†	0.87 ± 0.01^a^†	0.91 ± 0.02#
IVSs (mm)	0.81 ± 0.01	0.89 ± 0.01*	0.75 ± 0.03	0.86 ± 0.01	0.98 ± 0.02†	1.06 ± 0.03†	0.89 ± 0.01^a^†	0.93 ± 0.02#
LVIDs (mm)	2.10 ± 0.04	1.34 ± 0.07*	2.37 ± 0.13	1.38 ± 0.10*	1.56 ± 0.09†	0.80 ± 0.07^a^†	2.12 ± 0.15^a^	1.29 ± 0.11#
LVPWs (mm)	0.83 ± 0.01	0.90 ± 0.01	0.78 ± 0.03	0.89 ± 0.01	1.02 ± 0.03†	1.13 ± 0.05^a^†	0.91 ± 0.01	0.96 ± 0.02#
EDV (ml)	0.099 ± 0.005	0.082 ± 0.007	0.109 ± 0.012	0.066 ± 0.006*	0.071 ± 0.006†	0.044 ± 0.003^a^†	0.101 ± 0.013^a^	0.058 ± 0.009
ESV (ml)	0.025 ± 0.001	0.007 ± 0.001*	0.036 ± 0.006*	0.008 ± 0.001*	0.012 ± 0.002†	0.002 ± 0.001^a^	0.026 ± 0.005^a^	0.006 ± 0.002
EF (%)	74.5 ± 0.9	91.7 ± 0.7*	67.3 ± 2.0*	88.5 ± 1.8*	83.6 ± 2.0†	96.5 ± 0.8^a^	74.7 ± 2.0^a^	89.4 ± 1.4
FS (%)	37.6 ± 0.5	57.6 ± 1.3*	32.1 ± 1.5	53.0 ± 3.0*	47.1 ± 2.1†	68.9 ± 2.0^a^†	37.8 ± 1.7^a^	53.9 ± 2.1#
SV (ml)	0.074 ± 0.003	0.075 ± 0.006	0.072 ± 0.006	0.059 ± 0.005*	0.058 ± 0.004†	0.043 ± 0.003^a^†	0.075 ± 0.008	0.052 ± 0.008
RWT	0.48 ± 0.01	0.57 ± 0.03	0.43 ± 0.02	0.58 ± 0.02	0.66 ± 0.03†	0.81 ± 0.04^a^†	0.52 ± 0.03^a^	0.66 ± 0.04#

Echocardiography parameters recorded on wt and αMHC^403/+^ mice following in vivo treatment of 2 × 20 mg/kg isoproterenol in the absence or the presence of 1 mg/kg atenolol (Ate). IVSd—intraventricular septal wall thickness at end diastole, LVIDd—left ventricular internal diameter end diastole, LVPWd—left ventricular posterior wall end diastole, IVSs—intraventricular septal wall thickness at end systole, LVIDs—left ventricular internal diameter end systole, LVPWs—left ventricular posterior wall end systole, EDV—end-diastolic volume, ESV—end systolic volume, EF (%)—ejection fraction, FS (%)—fractional shortening, SV—stroke volume, RWT—relative wall thickness. Values are means ± SEM. Brown-Forsyth and Welch ANOVA was used to analyze differences between wt and αMHC^403/+^ mice followed by a Dunn’s test to correct for multiple comparisons. *p < 0.05 versus ctrl wt, ^a^*p* < 0.05 versus ctrl αMHC^403/+^, †*p* < 0.05 wt versus αMHC^403/+^ under the same condition, #p < 0.05 versus ISO αMHC^403/+^.

To further investigate the effect of ISO, mice were pre-treated for 10 min with atenolol, a selective β1 AR antagonist. Low dose (1 mg/kg, i.p.) atenolol significantly reduced the heart rate in both wt and αMHC^403/+^ mice (Fig. 1I,J,O) but the effect was more pronounced in the mutant mice (Table 1). As expected, selective β-blocker pretreatment also relaxed the left ventricles (Table 2, Fig. S1F,H vs. E,G). ISO increased the heart rate in the presence of atenolol in both wt and αMHC^403/+^ mice (Fig. 1K and Table 1), but importantly heart rate did not remain elevated in the mutant mice treated with atenolol after 2 h’ recovery (Table 1, Fig. 1L,O). The reduction in R amplitude and increase in the relative occurrence of arrhythmic events was less pronounced in the presence of the β_1_-AR blocker in αMHC^403/+^ mice (Table 1, Fig. 1O). The recovery from in vivo ISO treatment when atenolol was present was similar to wt mice (Table 1, Figs. 1L,O, S1I,J). αMHC^403/+^ mice were bright, alert, mobile and active similar to wt mice when pre treated with atenolol followed by ISO. Our data confirm that in addition to facilitating ventricular filling, cardiac selective β1AR blockers can reduce arrhythmogenic activity in αMHC^403/+^ hypertrophic hearts and decrease lethargy post sympathetic nervous system stimulation.

### αMHC^403/+^ ventricular myocytes exhibit prolonged action potential duration that shortens in the presence of ISO

**Figure 2:**
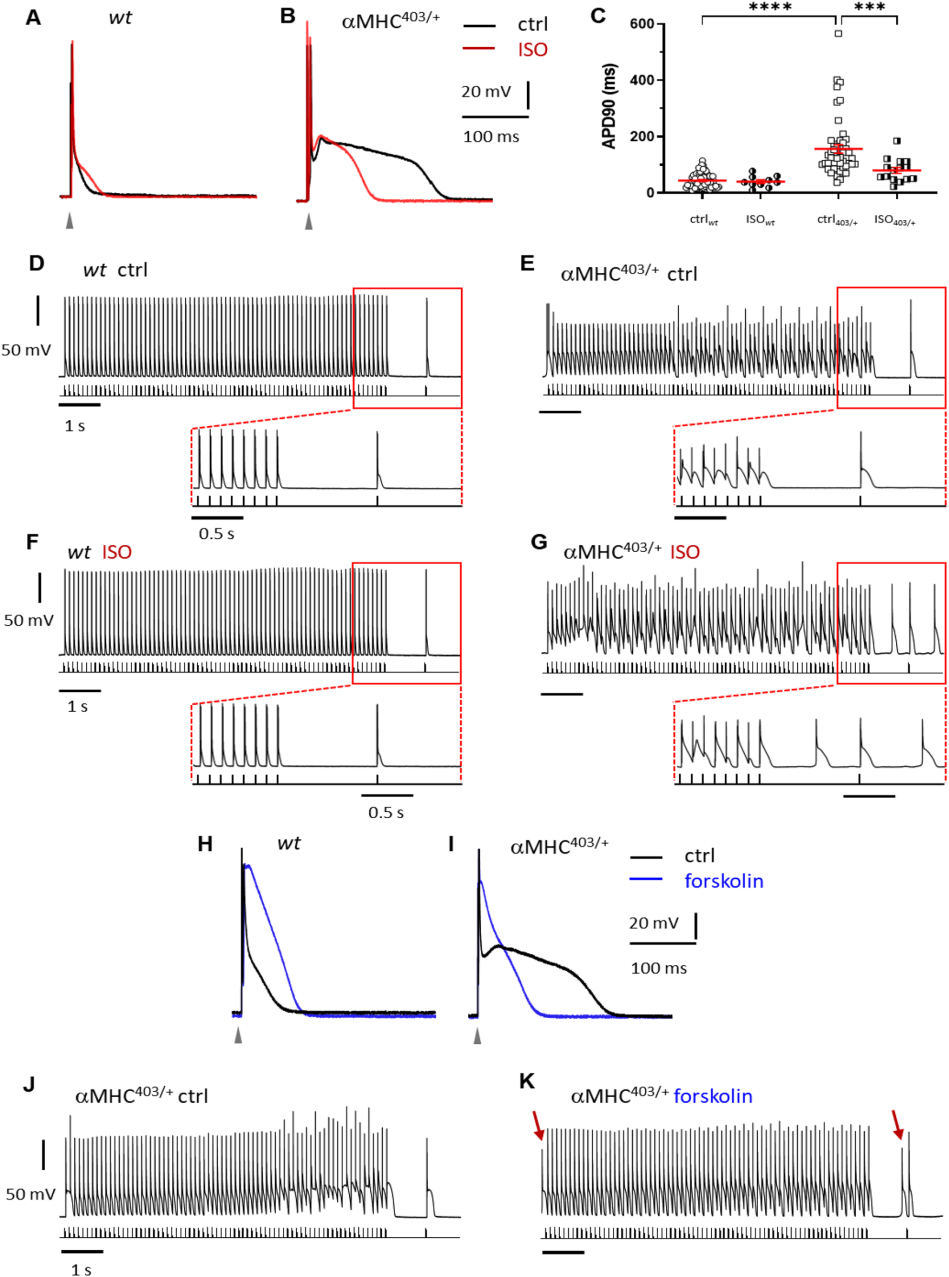
Representative AP recordings from wt (**A**) and αMHC^403/+^ ventricular myocytes (**B**), in the absence (black lines) or presence of 100 nM ISO (red). AP durations at 90% repolarization in the absence (wt n = 62, N = 21; αMHC^403/+^ n = 63, N = 19), or presence of isoproterenol (wt n = 10, N = 7; αMHC^403/+^ n = 17, N = 5) (**C**). Representative AP train recordings (9 Hz) on wt (**D** and **F**) and αMHC^403/+^ cardiac myocytes (**E** and **G**), under control conditions (**D**, **E**) or the presence of 100 nM ISO (**F**, **G**). Zoomed area shows the last 3 s of the 10 s recordings. Representative AP recordings from wt (**H**) and αMHC^403/+^ cardiac myocytes (**I**), in the absence (black) or presence of 10 μM forskolin (blue). Representative AP train recordings in control conditions (**J**) or the presence of 10 μM forskolin (**K**) on αMHC^403/+^ cardiac myocytes. Arrows indicate DADs.

Next we assessed the AP characteristics of cardiac myocytes isolated from adult hypertrophic αMHC^403/+^ mice in the absence and presence of acute exposure to ISO (100 nM). Under control conditions, at 1 Hz the resting membrane potential of αMHC^403/+^ myocytes was slightly depolarized and AP duration significantly prolonged (APD90: 165.1 ± 12.7 vs. 47.2 ± 4.1; n = 62 and n = 63, respectively) (Fig. 2A–C and Table 3). No significant difference in the amplitude of the AP was recorded. Consistent with this the expression level of the cardiac sodium channel protein Na_V_1.5 was unchanged in αMHC^403/+^ hearts (Fig. S2). wt cardiac myocytes showed no triggered spontaneous automaticity, while 88.2% of αMHC^403/+^ cardiac myocytes developed triggered activity, and delayed afterdepolarizations (DADs) at low (1 Hz) stimulation frequency (Table 3). Exposure to ISO caused a prolongation in action potential duration in wt cardiac myocytes (APD50 and APD90, Fig. 2A, Table 3) but *shortened* αMHC^403/+^ myocyte APD90 (Fig. 2B,C and Table 3). ISO also significantly increased the probability of DADs in αMHC^403/+^ myocytes (Fig. 2E,G, Table 3 and Fig. S3).

**Table 3:** Action potential parameters of wt and αMHC^403/+^ ventricular myocytes and frequency of delayed afterdepolarizations.

Action potential parameters	wt			αMHC^403/+^			
	ctrl	100 nM ISO	10 μM forskolin	ctrl	100 nM ISO	3 μM PKI + 100 nM ISO	10 μM forskolin
n	62	10	6	63	17	12	10
AP amplitude (mV)	93.1 ± 3.0	76.2 ± 9.9	97.5 ± 16.0	85.5 ± 3.1	105.6 ± 7.8	81.7 ± 5.2	87.4 ± 6.7
resting membrane potential (mV)	− 69.8 ± 0.6	− 72.4 ± 1.2	− 68.6 ± 3.7	− 66.2 ± 0.7*	− 62.3 ± 1.7^a^	− 67.2 ± 1.0	− 73.4 ± 1.4^b$^
APD50	4.8 ± 0.5	6.2 ± 1.8	12.0 ± 5.7	8.8 ± 1.9	5.1 ± 1.4	16.6 ± 4.1^b$^	37.0 ± 9.3^b$^
APD90	47.2 ± 4.1	49.2 ± 8.0	72.9 ± 7.1*	165.1 ± 12.7*	107.7 ± 32.7^b^	129.1 ± 8.2^$^	131.1 ± 15.2
DAD frequency (1/s)	0.002 ± 0.001	0.004 ± 0.004	0.229 ± 0.190*	0.270 ± 0.073*	0.525 ± 0.192^b^*	0.216 ± 0.116	1.158 ± 0.313^b^

Action potential parameters recorded on wt and αMHC^403/+^ ventricular myocytes in the presence of 100 nM isoproterenol, in the presence or absence of 3 μM myristoylated protein kinase A inhibitor-(14–22)-amide (PKI), or 10 μM forskolin. APD50, APD90 are action potential durations at 50% or 90% repolarization, respectively; DAD frequency: delayed afterdepolarizations recorded at 1 Hz pacing frequency in current clamp mode. Values are means ± SEM, (n) number of cells. *Data were analyzed by 2 Way ANOVA followed by a Tukey’s test for multiple comparisons *p* < 0.05 versus wt ctrl, $*p* < 0.05 versus100 nM ISO αMHC^403/+ a^*p* < 0.05 versus wt 100 nM ISO ^b^*p* < 0.05 versus ctrl αMHC^403/+^.

It is well known that APD depends on heart rate or stimulation frequency^15^. Pacing αMHC^403/+^ ventricular myocytes at their baseline heartbeat frequency (9 Hz or 540 beats/min) revealed irregular AP patterns. At the high frequency the cycle lengths of the impulses were shorter than the triggered APD, resulting in ineffective repolarization, early afterdepolarizations (EADs) and consequently depolarized resting membrane potential (Fig. 2E and inset vs. wt control on Fig. 2D). ISO had no effect on the pacing pattern at low stimulation frequency (Fig. S3B), but high frequency stimulus aggravated the effect of ISO in αMHC^403/+^ cardiac myocytes (Fig. 2G). Furthermore ISO induced DADs in the αMHC^403/+^ cardiac myocytes, at all stimulation protocols (at 9 Hz in Fig. 2G vs. F and at 3 Hz showed on right panel in Fig. S3A vs. B). A long APD also results in a long refractory period, leading to impaired impulse conduction and reentry in the heart^15^. Overall, these data indicate that ISO increased the excitability and induction of arrhythmias in αMHC^403/+^ myocytes.

To further explore the role of the β-adrenergic receptor pathway, we applied the adenylyl cyclase activator forskolin and recorded changes in AP configuration during current clamp. DAD frequency increased in current clamped cardiac myocytes (1.158 ± 0.313 vs. 0.270 ± 0.073, Table 3 and Fig. 2J,K) and altered the AP characteristics compared to ISO (Fig. 2H,I and Table 3). Both forskolin and ISO shortened the AP in αMHC^403/+^ myocytes, but forskolin also significantly increased the APD50 (Fig. 2I). Isoproterenol did not alter the APD50, but shortened APD90, indicating a predominant effect on repolarization (Phase 3).

To confirm that ISO was increasing susceptibility to arrhythmias in αMHC^403/+^ ventricular myocytes via protein kinase A, we pre-treated cells for 30 min with a cell-permeable protein kinase A inhibitor PKI (myristoylated PKI 14–22 amide, Tocris). 3 μM PKI attenuated the arrhythmogenic effect of ISO, and DAD frequency in αMHC^403/+^ myocytes in the presence of 100 nM ISO (0.216 ± 0.116 + PKI vs. 0.525 ± 0.0192 *p* < 0.05 Table 3). Isoproterenol, in the presence of the protein kinase A inhibitor only slightly shortened the APD (Table 3). Our results indicate that PKA phosphorylation activated by the β-adrenergic signaling cascade is responsible for increased arrhythmic activity in mutant ventricular myocytes.

### Cardiac myocytes isolated from αMHC^403/+^ mice exhibit distinct electrophysiological features in the absence and presence of ISO

**Figure 3:**
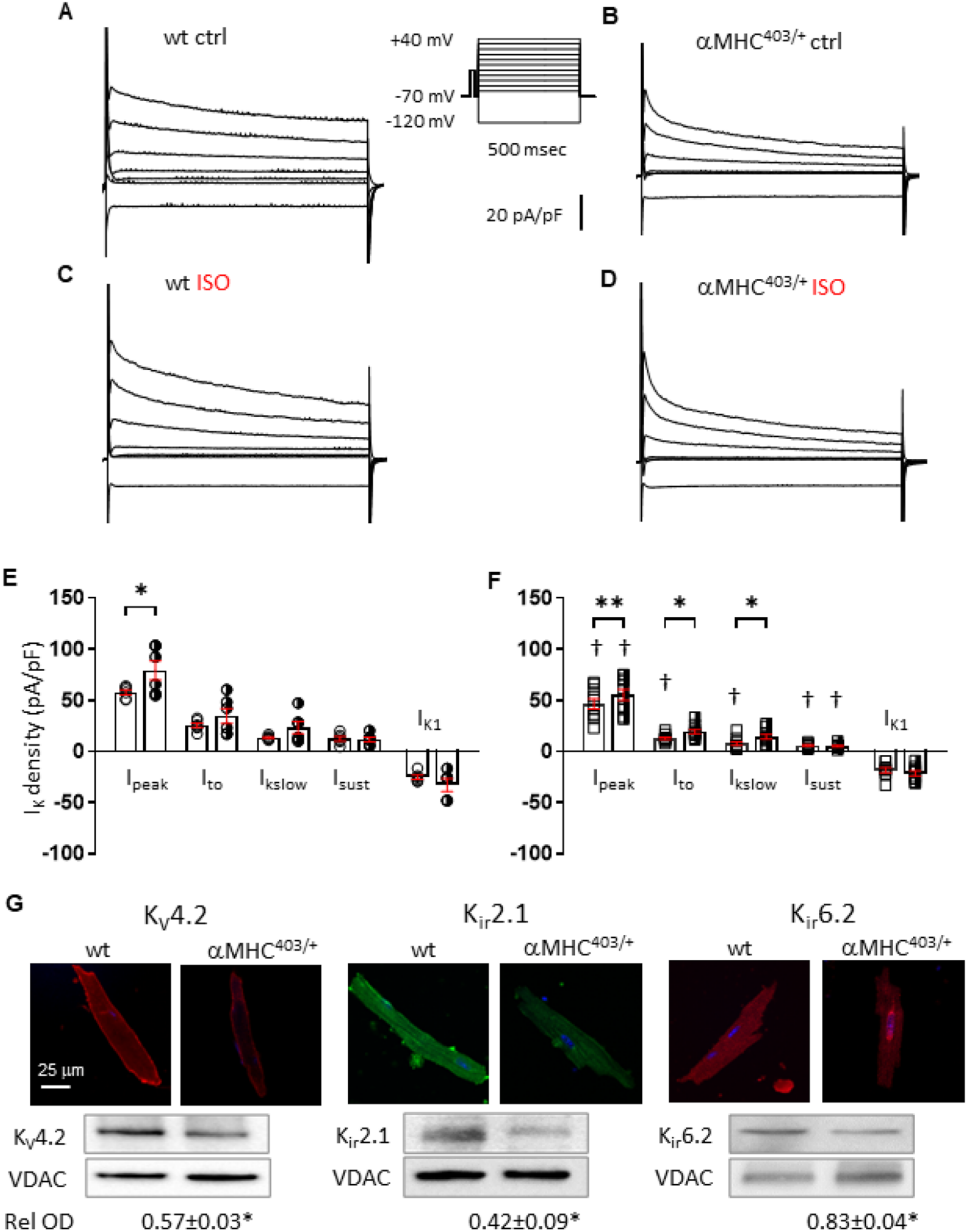
Representative potassium current recordings from wt (**A**, **C**) and αMHC^403/+^ (**B**, **D**) ventricular myocytes under control conditions (**A**, **B**) or the presence of 100 nM ISO (**C**, **D**). Whole-cell voltage-gated outward K^+^ (K_V_) currents were evoked in response to 500 ms depolarizing voltage steps to test potentials between − 60 and + 40 mV, in 10 mV increments, from a holding potential of − 70 mV (− 70, 0 mV and positive test potentials shown). Inward rectifying K^+^ currents (I_K1_) evoked in response to hyperpolarization to − 120 mV. (**E**–**F**) Scatter plot with bar graphs show I_K_ density (pA/pF) values for different K_V_ current components as means ± SEM. wt n = 8–15, N = 5 αMHC^403/+^ n = 10–16 N = 5*p < 0.05 control versus ISO, ^†^*p* < 0.05 wt versus αMHC^403/+^ (**G**) Representative immunofluorescence and corresponding Western blot images for K_V_4.2, K_ir_2.1, K_ir_6.2 potassium channels from wt and αMHC^403/+^ hearts as indicated. Relative optical density (Rel OD) values were calculated using VDAC (voltage dependent anion channel) as loading control. For further details please see section “Materials and methods”.

Arrhythmia formation in mouse cardiac myocytes can occur as a result of alterations in potassium or calcium currents^22^. Significant decreases in I_to_, I_Kslow_ and I_sust_ components of repolarizing potassium currents have been previously reported^18^ in pre-hypertrophic αMHC^403/+^ cardiac myocytes. In this study we recorded decreases in I_peak,_ I_to_, I_Kslow_ and I_sust_ components of the repolarizing potassium currents in left ventricular myocytes isolated from hypertrophic hearts (Fig. 3A vs. B,F vs. E). Corresponding with the electrophysiological changes, significantly decreased expression levels of K_V_4.2, K_ir_2.1 and K_ir_6.2 channel proteins were detected in αMHC^403/+^ versus wt hearts. Localization of the ATP-sensitive K^+^ channel K_ir_6.2 was also altered in αMHC^403/+^ myocytes (Fig. 3G). The expression and localization of other potassium channel proteins: K_V_1.4, K_V_1.5, K_V_2.1, K_V_11.1 and K_V_7.1, or their auxiliary subunit proteins TASK1, and KCNE1/MinK were not significantly altered in αMHC^403/+^ hearts (Fig. S2).

Interestingly αMHC^403/+^ cardiac myocytes exhibited a significant increase in sensitivity of I_to_ and I_Kslow_ currents to 100 nM ISO (Fig. 3D,F) versus wt myocytes (Fig. 3C,E). Reduced repolarization reserve and increased sensitivity of I_to_ and I_Kslow_ to ISO may contribute to the APD shortening observed in αMHC^403/+^ cardiac myocytes (Fig. 2B,C). Nevertheless this would only explain in part the increased arrhythmogenicity during sympathetic activation.

**Figure 4:**
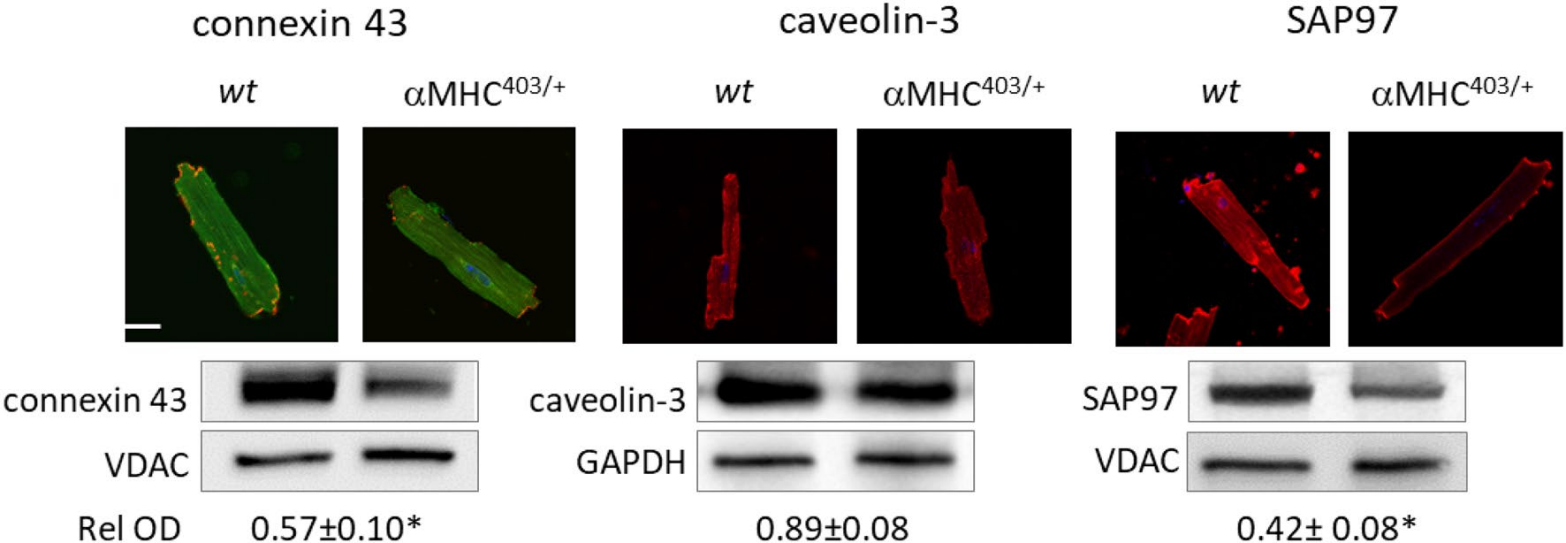
Representative immunofluorescence images of ventricular myocytes isolated from wt and αMHC^403/+^ hearts, immunolabelled with connexin 43, caveolin-3 and SAP97. Scale represents 20 μm. Representative Western blot images with relative optical density values are also shown for the same proteins, along with corresponding loading controls. *p < 0.05 wt versus αMHC^403/+^. For further detail see section “Materials and methods”.

**Figure 5:**
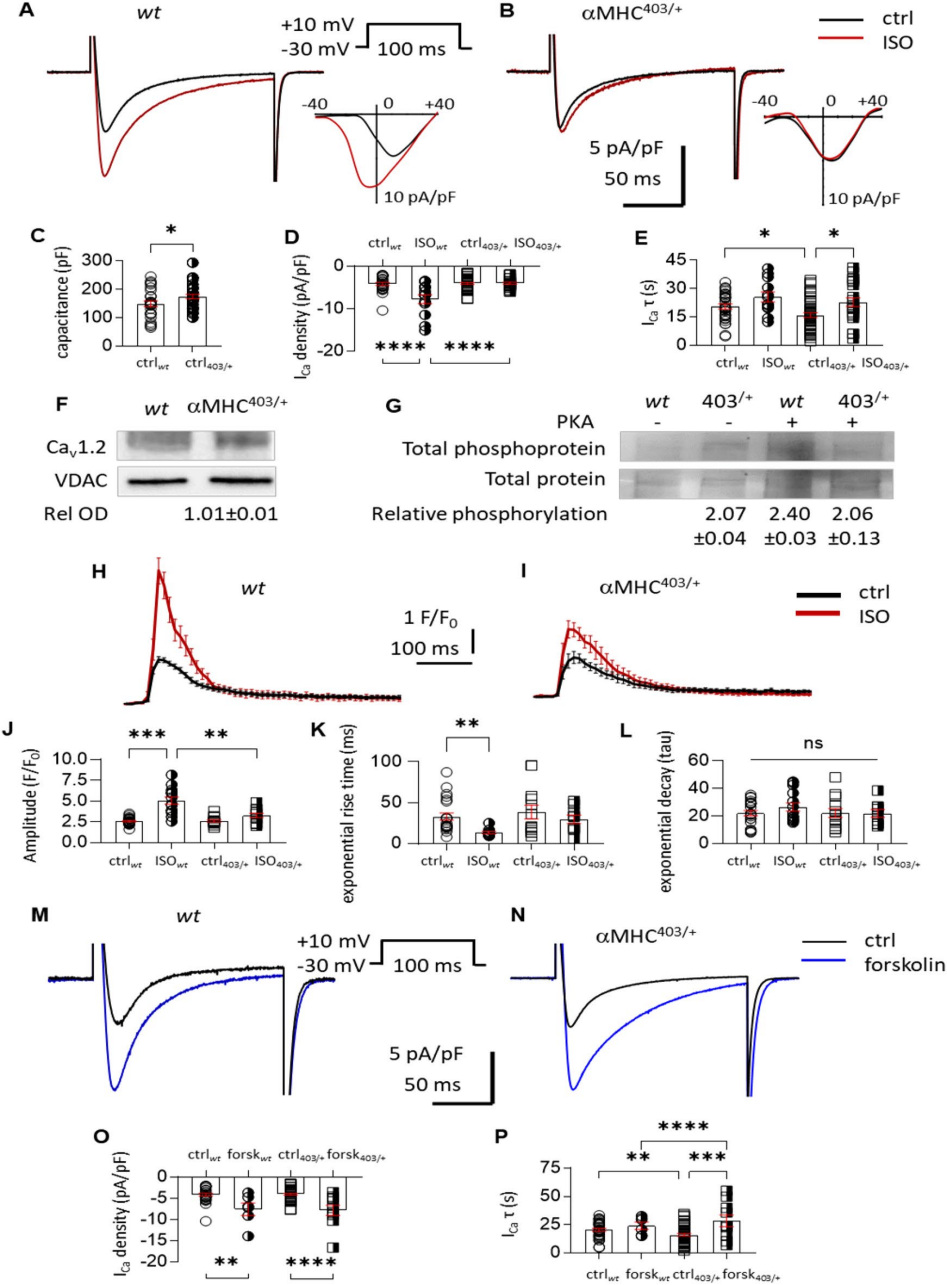
Representative calcium current recordings and IV traces from wt (**A**) and αMHC^403/+^ (**B**) ventricular myocytes, under control conditions (black lines) or the presence of ISO (red). Scatter plot with bar graphs show the cell capacitance (**C**) used to calculate I_Ca_ density (pA/pF) (**D**); and (**E**) the rate of inactivation (tau). Number of cells used for the study: wt n = 26, with ISO n = 13; αMHC^403/+^ n = 42, with ISO n = 22. (**F**) Representative Western blot images of Ca_V_1.2 protein using total heart homogenates from wt (N = 5) and αMHC^403/+^ hearts (N = 4) repeated in triplicate. (**G**) In vitro PKA phosphorylated immunoprecipitated protein samples were used to fluorescently detect total phosphoprotein as well as PKA-specific phosphorylation. (**H**, **I**) Representative Fluo-4 calcium transients acquired on current clamped ventricular myocytes isolated from wt (**H**) and αMHC^403/+^ mice (**I**) under control conditions (black traces, n = 23, N = 5; and n = 18, N = 5 respectively) or the presence of 100 nM ISO (red traces, (n = 13, N = 5; and n = 11, N = 5 respectively). Scatter plots with bar graphs show relative amplitude (as F/F_0_, **J**), exponential rise time (ms) (**K**) and exponential decay (tau) (**L**). (**M**–**N**) Representative calcium current recordings from wt (**M**) and αMHC^403/+^ (**N**) cardiac myocytes under control condition (black) or the presence of 10 μM forskolin (blue). Scatter plot with bar graphs show I_Ca_ density (pA/pF) (**O**); and the rate of inactivation (tau) of I_Ca_ traces (**P**) presented as means ± SEM *p < 0.05 control versus ISO, or wt versus αMHC^403/+^, number of asterisks representing increasing significance in *p* value. Number of cells used: wt n = 30 ctrl, n = 6 with forskolin and αMHC^403/+^ n = 45 ctrl, n = 10 with forskolin.

It is well recognised that Synapse Associated Protein 97 (SAP97) co-localises with K_ir_2 and K_V_ channel proteins, anchoring them to the plasma membrane, and aiding correct folding and function^23^. In addition to AKAP proteins, SAP97 participates in β1-adrenergic receptor localization and PKA phosphorylation^24^. We measured a significant decrease in SAP97 protein expression in cardiac tissue and myocytes isolated from αMHC^403/+^ mice versus wt mice (Fig. 4). Furthermore confocal imaging revealed differences in SAP97 protein localization with a relatively preserved surface membrane presentation of the protein, and reduced intracellular SAP97 content. Our data (Figs. 3G, 4) are consistent with changes in cell size and myofilament organization that are characteristic when structural changes such as hypertrophy are present. We measured a profound decrease (~ 40%) in connexin 43 protein expression in αMHC^403/+^ hearts (Fig. 4). This likely contributes to altered conductivity causing impulse propagation heterogeneity which in combination with decreased repolarization and impaired calcium handling provides a substrate for reentry arrhythmias^15,22^. Altered calcium handling in the αMHC^403/+^ model has been reported as a consequence of reduced SR calcium content and calcium accumulation by mutant myofilaments^11,16,21^. However systolic and diastolic calcium concentrations measured in wt and hypertrophic αMHC^403/+^ hearts are similar^17^. Consistent with our previous findings^7^, in the absence of ISO, quiescent adult αMHC^403/+^ cardiac myocytes demonstrated a small, but significant decrease in the kinetics of calcium current inactivation (Fig. 5B vs. 5A and E) of the L-type calcium channel (I_Ca_), with no change in peak amplitude (Fig. 5A,B), current density (Fig. 5D), activation or deactivation assessed as the integral of the current (Fig. S4A–C). As expected cell capacitance was increased in αMHC^403/+^ myocytes consistent with a hypertrophic phenotype (Fig. 5C). Immunoblot studies indicated that there was no significant difference in Ca_V_1.2 protein expression in heart homogenates from αMHC^403/+^ versus wt mice (Fig. 5F).

However, most surprisingly acute ISO exposure (100 nM) had no effect on current density (Fig. 5D), activation or deactivation parameters of I_Ca_ in αMHC^403/+^ (Fig. S4A–C), although it substantially increased I_Ca_ in wt cardiac myocytes (Figs. 5D,E, S4A–C). Under paced conditions (1 Hz), no significant difference in calcium transients was recorded in αMHC^403/+^ versus *wt* myocytes (Fig. 5H–L), in agreement with previous reports^17^. But in the presence of 100 nM ISO, the peak amplitude of the calcium transient was increased significantly in wt myocytes only (Fig. 5H, J). We examined the phosphorylation levels of immunoprecipitated Ca_V_1.2 protein and found increased basal phosphorylation in αMHC^403/+^ hearts. Exposure of the immunoprecipitated Ca_V_1.2 to PKA increased the phosphorylation level of the Ca_V_1.2 channel in wt but not αMHC^403/+^ hearts (Fig. 5G), suggesting that Ca_V_1.2 protein was already significantly phosphorylated in αMHC^403/+^ hearts under basal conditions. This was consistent with the lack of response of I_Ca_ to ISO in αMHC403 myocytes.

## αMHC^403/+^ myocytes exhibit altered Ca_V_1.2 and β1AR clustering and co-localization

In vitro electrophysiological and ex vivo biochemical studies demonstrated no difference in Ca_V_1.2 (Fig. 5F) and β1-adrenergic receptor expression (Fig. S2), or basal calcium current in αMHC^403/+^ versus *wt* hearts under control conditions (Fig. 5A,B,D). Recent studies have demonstrated super-clustering of Ca_V_1.2 promoted by β1-adrenergic receptor stimulation in mouse cardiac myocytes^25^. To investigate a potential role for altered channel clustering and Ca_V_1.2– β1-AR colocalization, we performed super resolution microscopy experiments.

**Figure 6:**
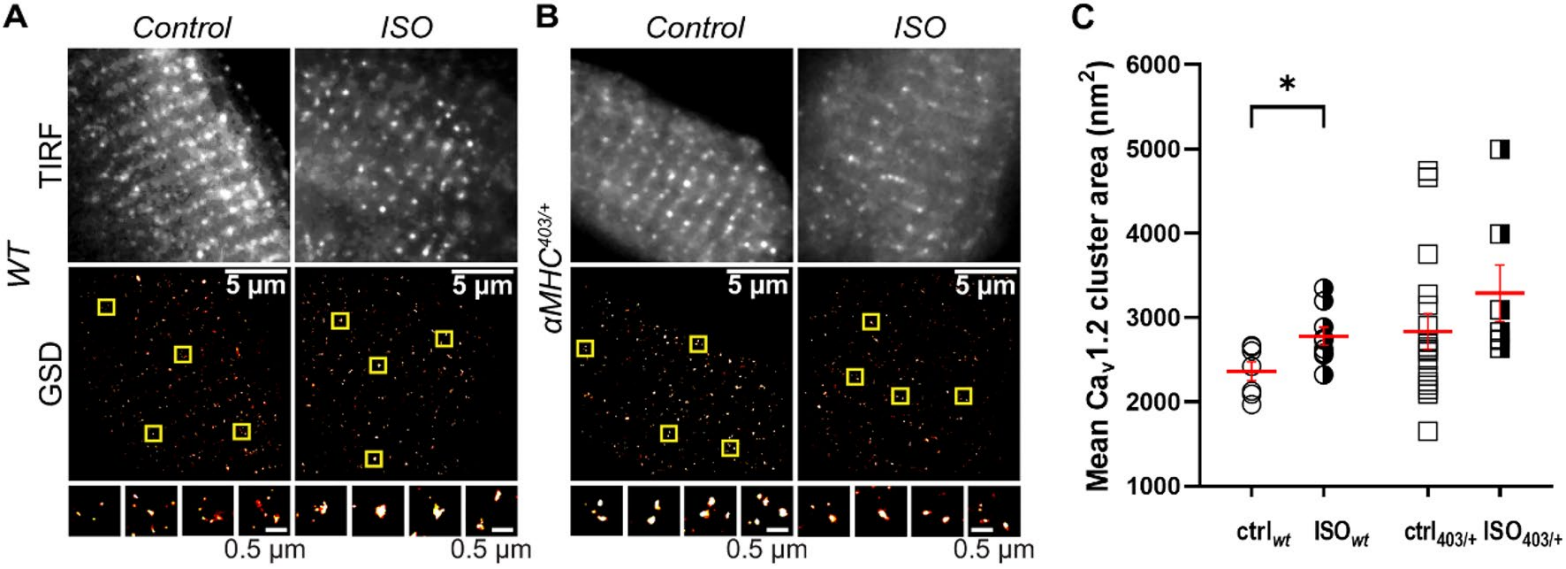
ISO-induced super-clustering response of Ca_V_1.2 in ventricular myocytes. (**A**–**B**) TIRF images (top row) and super-resolution GSD localization maps (bottom row) of immunostained Ca_V_1.2 channels in fixed adult ventricular myocytes isolated from *wt* (**A**) and αMHC^403/+^ mice (**B**). Control (left column) and ISO-stimulated (right column) myocytes are displayed side-by-side for comparison. Cluster ROIs indicated by yellow boxes in the GSD images appear magnified below the relevant image (bottom row). Mean Ca_V_1.2 channel cluster areas ± S.E.M. (indicated by red lines and error bars) are summarized for each condition in the aligned dot-plot (**C**). *p < 0.05 for comparisons as indicated. n = 14–27 ventricular myocytes, from N = 4–7 mice.

**Figure 7:**
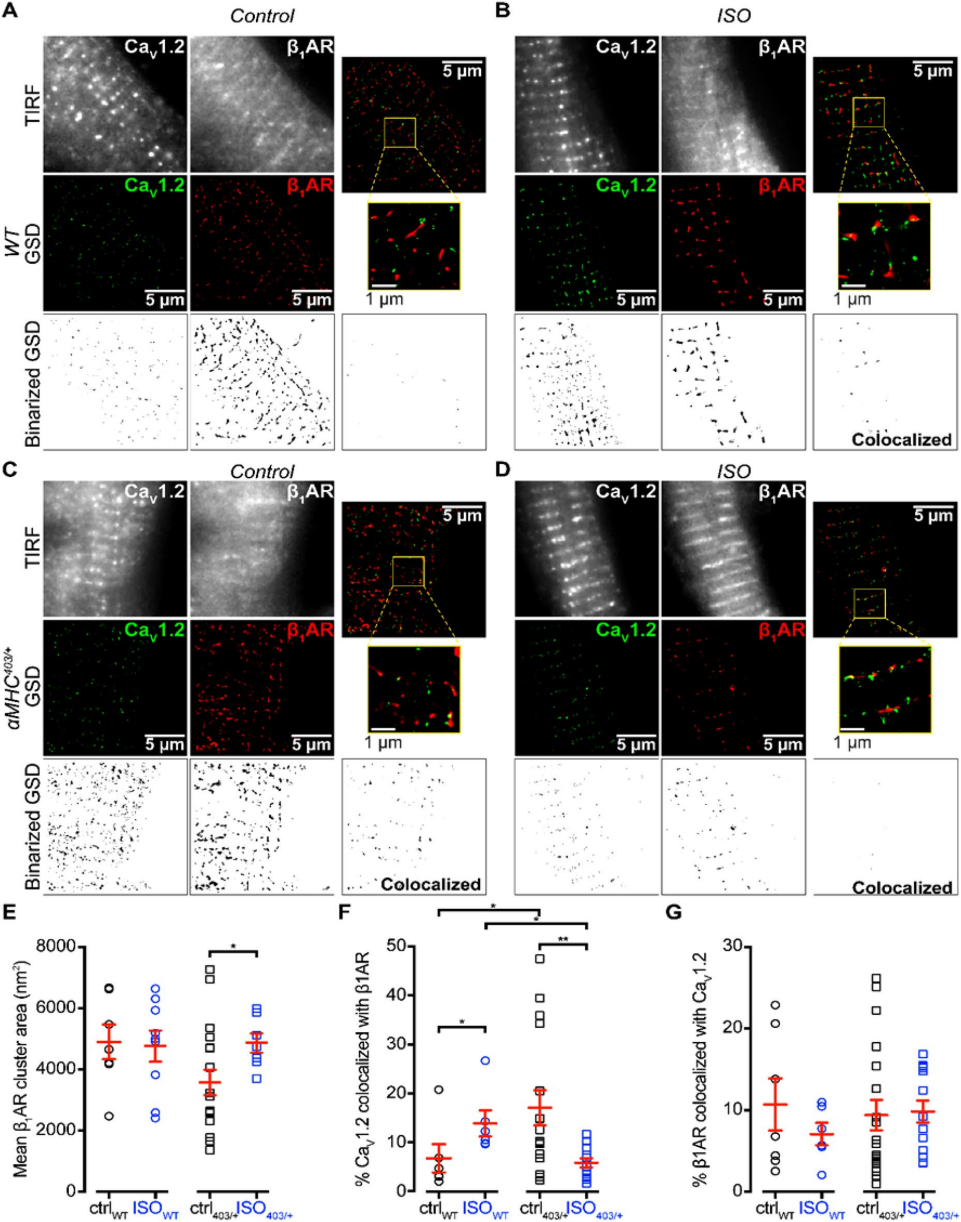
Colocalization of β1ΑRs with CaV1.2 is altered in αMHC^403/+^ mutant myocytes. (**A**) and (**B**): TIRF images (top row), super-resolution GSD localization maps (middle row) and binarized images (bottom row) of immunostained CaV1.2 channels and β1ARs in representative fixed adult ventricular myocytes isolated from WT mice under control (**A**) or ISO-stimulated conditions (**B**). Merged two-channel image showing relative distributions of CaV1.2 and β1ARs is shown in the third column with the colocalized binarized image. (**C**, **D**) Same layout format for myocytes isolated from αMHC^403/+^ mice. (**E**) Mean β1ARs cluster areas ± S.E.M. (indicated by red lines and error bars) are summarized for each condition in the aligned dot-plot. (**F**) % colocalization of CaV1.2 with β1ARs and G: β1ARs and CaV1.2 are summarized**.** *p < 0.05 for comparisons as indicated. n = 14–27 ventricular myocytes, from N = 4–7 mice.

Pre-treatment with ISO (100 nM isoproterenol, 10 min) induced a significant increase in the formation of superclusters of Ca_V_1.2 in wt but not in αMHC^403/+^ myocytes (Fig. 6)). While β1-AR cluster area was not significantly different in αMHC^403/+^ cardiac myocytes compared to wt myocytes under control conditions (Fig. 7A,C,E), ISO treatment significantly altered β1AR cluster area size in αMHC^403/+^, but not in wt myocytes (Fig. 7B,D,E).

Colocalization analysis of αMHC^403/+^ and *wt* cardiac myocytes labelled with Ca_V_1.2 and β1AR antibodies revealed a relatively small number of Ca_V_1.2 colocalized with β1ARs in wt cardiac myocytes and a significantly higher proportion of the Ca_V_1.2 colocalized with β1ARs in αMHC^403/+^ myocytes under control conditions (Fig. 7A–D,F). ISO treatment resulted in more Ca_V_1.2 protein colocalizing with β1ARs in wt cardiac myocytes, but under the same conditions, comparatively less Ca_V_1.2 were found to be localized with β1ARs in αMHC^403/+^ myocytes (Fig. 7A–D,F). Colocalization of the Ca_V_1.2 with the β1AR may explain the higher phosphorylation level of the channel protein in αMHC^403/+^ cardiac myocytes (Fig. 5G). There was no change in β1AR co-localizing with Ca_V_1.2 (Fig. 7A–D,G).

Previous studies have demonstrated a role for caveolin-3 in forming macromolecular complexes with β-adrenergic receptors and in β-adrenergic signaling^26,27^. Although αMHC^403/+^ myocytes displayed a reduced level of caveolin-3 expression (that was not statistically significant) versus wt myocytes (Fig. 4), no alterations in cluster area or colocalization with Ca_V_1.2 protein were observed (Fig. S5).

Our super resolution study indicated that Ca_V_1.2 protein and β-adrenergic receptor localization are altered in αMHC^403/+^ myocytes. To further confirm the altered Ca_V_1.2-β1AR co-localization, we examined the effect of forskolin on I_Ca_. In contrast to the lack of response to ISO, the addition of 10 µM forskolin significantly increased the amplitude, activation and inactivation of I_Ca_ in αMHC^403/+^ myocytes similar to that of wt myocytes (Figs. 5M–P, S4D–F). These data indicate that altered ion channel and β1AR localization are responsible for altered calcium handling in αMHC^403/+^ myocytes.

### Ca_V_1.2 is near maximally phosphorylated in αMHC^403/+^ mice

To further investigate the mechanisms for the poor recovery of αMHC^403/+^ mice following ISO treatment, hearts were collected following in vivo isoproterenol challenge, and phosphorylation of Ca_V_1.2 assessed. Untreated αMHC^403/+^ mouse hearts demonstrated phosphorylation of both Ca_V_1.2 and total protein compared to wt, that remained unchanged following ISO treatment (Fig. S6A–C). These data suggest that Ca_V_1.2 is phosphorylated under control conditions in αMHC^403/+^ mice. Creatine kinase (CK) activity was also significantly increased in hearts from ISO treated αMHC^403/+^ mice versus wt mice suggesting ongoing myocardial injury (Fig. S6D).

## Discussion

Hypertrophic cardiomyopathy is characterized by disorganization of cytoskeletal proteins and myofibrils, myocyte remodeling, fibrosis and altered energy metabolism^10^. We and others have demonstrated that αMHC^403/+^ mice develop clinical features similar to those found in human disease at both cellular and whole heart level.^7,21^. Alterations in the electrical properties of the cardiac myocyte that occur in response to stressors such as increased adrenergic stimulation can contribute to the genesis of ventricular arrhythmias and lead to sudden cardiac death^9^. The objective of this study was to investigate the mechanisms for increased arrhythmogenic activity resulting from the human FHC disease causing mutation during sympathetic stimulation.

Our data indicate that AP characteristics in αMHC^403/+^ myocytes were significantly prolonged and contrary to responses in wt cells, the AP shortened under conditions of increased adrenergic stimulation. At normal murine heartbeat-frequency (9 Hz) in the presence of ISO, membrane repolarization was incomplete in αMHC^403/+^ myocytes, the resting membrane potential became more depolarized, resulting in more early and delayed afterdepolarizations. In addition the AP refractory period was prolonged, which is a recognized substrate for impaired impulse conduction and reentry in the heart^15^.

The most apparent difference between human and murine AP kinetics is in the plateau phase. In mouse heart depolarizing I_Ca_ is less pronounced, while repolarizing I_to_ and I_Kur_ are more prominent, and as a result, murine APs demonstrate a more rapid repolarization. Nevertheless similarities in structure, excitation–contraction coupling, recovery and propagation of excitation can be investigated at the molecular, cellular, tissue, organ, and whole-animal level in the mouse^22,28^. To clarify any differences in APs between mouse and man, we performed additional experiments and assessed the AP characteristics of hiPSC-CMs (see SI methods) from a hypertrophic cardiomyopathy patient carrying R403Q *MYH7* mutation (*MYH7* 403^/+^, see family pedigree, Fig. S7C). Action potential measurements were performed with the kinetic imaging cytometry (KIC) platform. Similar to the mouse mutant myocytes, the AP duration of *MYH7* 403^/+^ was significantly prolonged (Fig. S7B,E,F) versus the isogenic CRISPR corrected control, *MYH7* 403^/−^ (Fig. S7A,D,F). Also similar to the αMHC^403/+^ myocytes, application of 1 µM ISO significantly shortened *MYH7* 403^/+^ AP duration while ISO slightly prolonged the isogenic CRISPR corrected control AP. Therefore action potential alterations in hiPSC-CMs are similar to αMHC^403/+^ myocytes and electrical remodeling at the cardiac myocyte level occurs early in FHC. In support of this, a high incidence of arrhythmias and sudden cardiac death in cardiac troponin T (*TnT*-I79N) mutant mice have been reported, in the absence of a hypertrophic phenotype. Introducing the *TnT*-I79N mutation into human induced pluripotent stem cells with CRISPR/Cas9 technique reproduced key features of FHC including myofilament disarray, hypercontractility and diastolic dysfunction, as well as alterations in the ventricular AP^29^. However the proposed mechanisms often differ between specific mutations. In the *TNNT2*-R92Q mouse model^30^, a decrease in Na_V_1.5 and increase in Ca_V_1.2 expression and late sodium current (I_NaL_) contribute to the phenotype. Isoproterenol further prolongs the AP. In our model, we found no difference in Na_V_1.5 or Ca_V_1.2 expression and isoproterenol shortened the AP duration.

In addition to a decrease in the expression and function of some potassium channels, we report here an increase in the sensitivity of I_to_ and I_Kslow_ to ISO that appeared to contribute to the APD shortening observed in αMHC^403/+^ cardiac myocytes. SAP97 protein expression was decreased in mutant hearts consistent with an alteration in the trafficking and localization of K^+^ channels. SAP97 protein also localized to the β1-adrenergic signaling complex. Importantly we demonstrated a substantial decrease in connexin 43 protein expression that correlates with morphological and histological changes in the hypertrophic myocardium. This can contribute to impaired impulse conduction leading to impulse propagation heterogeneity^15,22^.

Consistent with previous studies, we found no difference in diastolic or systolic intracellular calcium levels in αMHC^403/+^ myocytes or in Ca_V_1.2 expression compared with wt hearts^7,17^. However, despite the small, but significant difference in the inactivation rate of the channel and calcium transients, we could not increase the Ca_v_1.2 current when αMHC^403/+^ myocytes were exposed to β1-adrenergic stimulation. Consistent with this we revealed an elevated phosphorylation state of the Ca_V_1.2 channel protein extracted from cardiomyopathic αMHC^403/+^ mice. We cannot rule out the possibility that altered phosphatase activity contributes to the elevated phosphorylation state of the channel.

It is well documented that β1-adrenergic stimulation enhances the cardiac L-type calcium channel activity. To facilitate co-operative gating the Ca_V_1.2 molecules form super-clusters^25^. Assessed with super-resolution microscopy we demonstrate that in ventricular myocytes isolated from hypertrophic αMHC^403/+^ hearts Ca_V_1.2 proteins form clusters, similar to wt cells, but isoproterenol treatment only increased the size of these clusters significantly in wt cardiac myocytes. The Ca_V_1.2 molecules not only clustered differently, but there were more Ca_V_1.2 localized together with β1AR’s in mutant myocytes. This demonstrates for the first time a direct interaction between Ca_V_1.2 and β1ARs, which can explain the higher phosphorylation level of the channel protein in αMHC^403/+^ cardiac myocytes. In vitro ISO treatment stimulated colocalization of Ca_V_1.2 and β1ARs in wt cardiac myocytes, but in αMHC^403/+^ myocytes it decreased the number of the Ca_V_1.2 localized with β1ARs.

Here we demonstrate that β-adrenergic stimulation alone is sufficient to increase the probability of arrhythmic activity in αMHC^403/+^ mice. Hypertrophic hearts of the αMHC^403/+^ mice not only demonstrated ISO-induced arrhythmias, but the mice did not recover well from the ISO challenge resulting in fatigue and tissue damage assessed as a significant increase in creatine kinase activity in the αMHC^403/+^ hearts but not wt hearts. FHC patients reportedly experience serious cardiovascular events and fatigue following vigorous exercise^12^.

Our results suggest that cytoskeletal disarray contributes to the alterations in ion channel and β1 adrenergic receptor localization and function in the αMHC^403/+^ heart. Reduced repolarization reserve and altered conduction velocity are associated with the generation of arrhythmias during β1-adrenergic receptor stimulation in the αMHC^403/+^ heart. However, pro-arrhythmic mechanisms may vary depending on the underlying gene mutation reinforcing the need to individualize treatment options with the genetic mutation. We find that inhibition of the β1AR with atenolol relaxed ventricular muscle and improved filling, but also significantly reduced the occurrence of arrhythmic events and allowed the mice to recover fully from the adrenergic challenge. Our data indicate that treatment with selective β1AR blockers may be sufficient to manage arrhythmias in patients carrying an R403Q mutation. Furthermore we highlight the significance of cytoskeletal disarray in altering ion channel location and function and β-adrenergic receptor signaling, leading to electrical instability in the FHC heart.

## Materials and methods

### Mouse model

Male 35–45 wk old heterozygous αMHC^403/+^ mice expressing the human disease-causing mutation R403Q in *MYH6* were used. We used male mice because female mice carrying the αMHCR403Q^/+^ mutation develop hypertrophic cardiomyopathy less consistently than males. Mice were used to establish a colony received as a gift from C and J Seidman (Department of Genetics, Harvard Medical School, MA). Negatively genotyped male age-matched littermates were used as wild type (wt) controls. A total number of 68 wt and 92 αMHC^403/+^ mice were used in the study. In the text N indicates number of animals, n indicates number of cells.

All experiments were approved by The Animal Ethics Committee of The University of Western Australia in accordance with the Australian Code of Practice for the Care and Use of Animals for Scientific Purposes (NHMRC, 8th Edition, 2013; updated 2021) and all methods reported in accordance with the ARRIVE guidelines.

### Electrocardiography and echocardiography studies

Mice were anesthetized using methoxyflurane and placed on a warming plate (37 °C). Electrocardiograms (ECGs) were recorded with s.c. bipolar leads (lead II) using a PowerLab data acquisition system with Animal BioAmp for 10 min prior to (control) and following i.p. isoproterenol (ISO, two doses of 20 mg/kg administered 10 min apart) or atenolol (1 mg/kg). Parameters were measured on signal-averaged complexes derived from 10 s of contiguous data. QT interval was corrected for heart rate using the Mitchell method^31^. The relative occurrence of arrhythmic events was measured as number of irregular beats per second of recording (LabChart ADInstruments).

In parallel experiments, echocardiograms were recorded using i13L probe on a Vivid 7 Dimension (GE Healthcare) as previously described^32^.

## Electrophysiology

### Action potential recordings

Left ventricular cardiac myocytes were isolated as described^32^, for more details please see SI. Cells were stimulated in current clamp mode at 1, 3 or 9 Hz with 0.2 ms suprathreshold stimuli. Glass pipettes (4–5 MΩ) were filled with pipette solution (in mM): 120 K-glutamate, 20 KCl, 10 NaCl, 2 MgCl_2_, 0.1 EGTA, 5 Hepes, 5 MgATP, 0.03 CaCl_2_, pH 7.05. Experiments were performed at 37 °C in Tyrode solution (in mM): 140 NaCl, 5.4 KCl, 1 CaCl_2_, 0.5 MgCl_2_, 5.5 Hepes, 11 glucose, pH 7.4 at room temperature. AP duration was measured at 90% (APD90) and 50% (APD50) of repolarization.

### Potassium (K^+^) and calcium (Ca^2+^) current recordings

For recording of whole-cell K^+^ currents, pipettes were filled with AP recording pipette solution. Bath solution contained (in mM): 136 NaCl, 4 KCl, 2 MgCl_2_, 1 CaCl_2_, 10 Hepes, 10 glucose, 0.02 tetrodotoxin (TTX, Tocris), 0.002 nisoldipine, pH 7.4.. Whole-cell voltage-gated K^+^ currents were evoked in response to 500 ms depolarizing voltage steps to test potentials of − 120 mV and between − 60 and + 40 mV (10 mV increments) from a holding potential of − 70 mV. Experiments were performed at 37 °C. Peak K_V_ current and I_K1_ amplitudes were measured as the maximal amplitudes of currents. I_to_ amplitude was determined from exponential fit to the decay phase of the outward K^+^ current, I_sustained_ component as the current amplitude at the end of the test pulse and I_K,slow_ was calculated as the difference between I_to_ and I_sustained_^18^.

Ca^2+^ currents were recorded in bath solution (in mM): 140 NaCl, 5.4 CsCl, 1 CaCl_2_, 0.5 MgCl_2_, 5.5 HEPES, 11 glucose, pH 7.4 at 37 °C as previously described^32^. Pipette solution contained (in mM): 115 CsCl, 1 CaCl_2_, 20 TEA-Cl, 10 HEPES, 10 EGTA, 5 MgATP, 0.1 Tris-GTP, 10 phosphocreatine, pH7.05. Ca^2+^ currents were monitored by applying a 100 ms test pulse to 10 mV after a 50 ms prepulse to − 30 mV once every 10 s. Kinetics of calcium current inactivation was analysed by fitting current decay after channel activation with a bi-exponential function (yielding tau 1 and tau 2). Current activation and inactivation were also assessed by calculating the integral or “area under a curve” as the area between the graph of *y* = *f(x)* and the *x*-axis.

Both APs and whole cell currents were recorded using an Axopatch 200B voltage-clamp amplifier (Molecular Devices) and an IBM compatible computer with a Digidata 1400 interface and pClamp10 software (Molecular Devices). Cardiac myocytes isolated from the same animal were used for AP and Ca^2+^ or K^+^ current measurements. Data analyses were executed with Clampfit10 and GraphPad Prism8, results are reported as mean ± SEM.

### Immunoblotting

Tissue was weighted, homogenised in 1:4 RIPA buffer, consisting of (in mM): 150 NaCl, 50 Tris, 20 Na_4_P_2_O_7_, 2 Na_3_VO_4_, 1 NaF, 0.5% Na deoxycholate, 1% Triton X-100, 0.1% SDS, EDTA-free Complete protease inhibitor cocktail (Roche), and Phosphatase inhibitor cocktail, pH7.4. The homogenate was centrifuged at 10,000 g for 5 min at 4 °C. 25 μg of pooled tissue homogenate (N = 3 wt and 3 αMHC^403/+^ hearts) was loaded into precast 10% Bio-Rad Mini-Protean TGX Stain-FreeTM SDS–polyacrylamide gel, then electrophoretically transferred to 0.2 µm nitrocellulose membrane (Trans-Blot TurboTM Transfer Pack (Bio-Rad) using the Bio-Rad Trans-Blot TurboTM Transfer System. Western blot experiments were run in triplicate, representative images are shown in figures. Antibodies used in the study are listed in SI. Densitometry was performed using ImageJ software. Background subtracted intensity values were normalized to loading controls VDAC or GAPDH signal on the same blot. For more details and for full length blots see SI.

### Immunprecipitation and in vitro phosphorylation of Ca_V_1.2 protein

Anti-Ca_V_1.2 antibody pre-incubated with Dynabeads Protein G (Thermo Fisher Scientific) used to pull out the Ca_V_1.2 protein from tissue homogenates. 2 Unit PKA catalytic subunit (Promega) was used per each μg of immunprecipitated protein to perform in vitro phosphorylation as previously described^33^. All immunoblot experiments were run as triplicate, representative images were shown on figures. For more details and for original blots see SI.

### Immunocytochemistry

Precision cover glass (No. 1.5H, Marienfeld Superior) cleaned then coated with poly-L-lysine (0.01%, 20 min) and laminin (20 μg/ml, 45 min; Life Technologies).

For confocal microscopy cells were fixed with 4% formaldehyde, solubilized with 0.5% TritonX-100. Primary antibodies, used for Western blots (1:100 dilution, 5% BSA, 2 h, RT) and fluorescently labelled (Alexa Fluor 488 or Alexa Fluor 555) secondary antibodies (1:1000, 1 h RT, Abcam) were used. After mounting with ProLong™ Glass Antifade Mountant (ThermoFisher), cells were imaged with Nikon C2 confocal microscope coupled with NIS elements software.

For super-resolution studies, some coverslip adherent cells were treated for 10 min with 100 nM isoproterenol prior to fixation in ice-cold methanol for, 5 min. After washing cells were blocked (45 min, RT) in blocking buffer: 20% SEA Block (Thermo Fisher Scientific), 0.05% v/v Triton X‐100 in PBS. Cells were incubated overnight at 4 °C with mouse monoclonal anti‐Ca_V_1.2 (UC Davis/NIH NeuroMab Facility, clone N263/31; 1:100) with rabbit polyclonal anti-caveolin-3 or rabbit polyclonal β1 adrenergic receptor antibody. Secondary antibodies used were Alexa Fluor 647‐conjugated goat anti‐mouse IgG2b or Alexa Fluor 555‐conjugated goat anti‐rabbit (Life Technologies, 1:1000).

### Super-resolution nanoscopy

Cells were imaged on a super-resolution Ground State Depletion (GSD) microscope (Leica Microsystems, Wetzlar, courtesy of Dr. F Santana) in TIRF mode with 150 nm penetration depth as previously described^34^. Fluorescence was detected through a Leica high-power TIRF quad filter cube (QGS HP-T) with emission band-pass filters. The collected frames were reconstructed into super-resolution localization maps using Leica Application Suite (LAS AF) software. Cluster area size was measured from binary masks of the localization maps with a 10 nm pixel size in ImageJ/Fiji as previously described^35^.

### Assessment of changes in intracellular calcium under paced conditions

Myocytes were incubated in Fluo-4-AM (5 μM, Life Technologies, 20 min), then stimulated in current clamp mode with suprathreshold stimuli using an Axopatch 200B voltage-clamp amplifier (Molecular Devices). Signal was recorded using a Zyla 5.5 sCMOS camera and MetaMorph 7.10.3 sotware.

### Statistical analysis

Results are reported as means ± SEM. Statistical analysis was performed using Prism GraphPad software. The Shapiro–Wilk normality test was used to assess whether the data were normally distributed. If the data were normally distributed a Brown-Forsyth and Welch ANOVA was used to analyse differences between wt and αMHC^403/+^ groups. Where data were not normally distributed a Kruskal–Wallis ANOVA was performed. A Dunn’s test was used to correct for multiple comparisons. All chemicals and reagents were purchased from Sigma-Merck unless otherwise specified.

## Supplementary Information

Supplementary Information.

## Data Availability

Datasets generated and/or analysed during the current study are available in The University of Western Australia Data Repository https://research-repository.uwa.edu.au/en/datasets/. Additional datasets used and/or analysed by collaborating authors during the current study are available from the corresponding author on reasonable request.
