# Supplementary material for: Cytoskeletal disarray increases arrhythmogenic vulnerability during sympathetic stimulation in a model of hypertrophic cardiomyopathy
Source: Sci Rep. 2023 Jul 12;13:11296. doi: 10.1038/s41598-023-38296-2 (PMC10338442; doi:10.1038/s41598-023-38296-2)
Supplement: Supplementary file 1 — Supplementary Information. [file 41598_2023_38296_MOESM1_ESM.pdf]

Ref: Submission ID 896f7808-7e8e-4dbf-b775-1894650fd0f5 R1

Supplementary Information

Cytoskeletal disarray increases arrhythmogenic vulnerability during sympathetic stimulation in a model of hypertrophic cardiomyopathy

Henrietta Cserne Szappanos, Helena M. Viola, Danica W. Ito, Seakcheng Lim, Melissa Mangala, Mira Holliday, Samantha Barratt Ross, Christopher Semsarian, Adam Hill, Rose E. Dixon, Livia C. Hool

Corresponding author: Livia C. Hool
Email: livia.hool@uwa.edu.au

This PDF file includes:

Supporting text
Figures S1 to S7
SI References

Other supporting materials for this manuscript include the following:

Original, unedited, representative full Western blot images and original unprocessed full length western blots

Supporting Text

SI Methods

Isolation of cardiac myocytes

Mice were anesthetized with methoxyflurane followed by 160 mg/kg pentobarbitone, i.p. as approved by The Animal Ethics Committee of The University of Western Australia in accordance with the Australian Code of Practice for the Care and Use of Animals for Scientific Purposes (NHMRC, 8th Edition, 2013; updated 2021).

Excised hearts were cannulated onto a Langendorff apparatus and perfused with Krebs-Henseleit Buffer (in mM): 120 NaCl, 25 $NaHCO_3$, 4.8 KCl, 2.2 $MgSO_4$, 1.2 NaH_2PO_4, 11 glucose (pH7.35 with O_2/CO_2 at 37°C). After enzymatic digestion with 1.4 mg/ml collagenase B (Worthington Biochemical Co), the cells were kept in calcium free Hepes-Buffered Solution (HBS) containing (in mM): 139 NaCl, 5.6 Na_2HPO_4, 5.3 KCl, 0.4 $MgSO_4$, 5 glucose, 20 Hepes, 2 glutamine (pH 7.4).

Immunoblotting

Primary antibodies used include: rabbit polyclonal anti-K_V1.4 (Alomone, #APC-167, 1:200), rabbit polyclonal anti-K_V1.5 (Alomone, #APC-004, 1:200), guinea pig polyclonal anti-K_V2.1 (Alomone, #AGP-109, 1:1000), rabbit polyclonal anti-K_V4.2 (Alomone, #APC-023, 1:200), guinea pig polyclonal anti-K_{ir}2.1 (Alomone, #AGP-044, 1:500), rabbit polyclonal anti-K_{2P}3.1 (TASK1) (Alomone, #APC-024, 1:200), rabbit polyclonal anti-K_{ir}6.2 (Alomone, # APC-020, 1:200), rabbit polyclonal anti-K_V11.1 (HERG) (Alomone, #APC-109-F, 1:200), rabbit polyclonal anti-KCNE1 (IsK, MinK) (Alomone, #APC-163, 1:500), rabbit polyclonal anti-KCNQ1 (K_V7.1) (Alomone, #APC-168, 1:500), rabbit polyclonal anti-SAP97 (ThermoFsher, #PA1-741, 1:500), rabbit polyclonal anti-Na_V1.5 (Alomone, #ASC-005, 1:500), rabbit polyclonal anti-Ca_V1.2 (Alomone, #ACC-003, 1:200), rabbit polyclonal anti-β_1 adrenergic receptor (Abcam, #ab3442, 1:1000), rabbit polyclonal anti-caveolin 3 (Abcam, #ab2912, 1:1000), rabbit polyclonal anti-connexin 43 (Cell Signalling, #3512, 1:1000), rabbit monoclonal anti-VDAC (Cell Signalling, #4661, 1:1000), rabbit monoclonal anti-GAPDH (Cell Signalling, #2118, 1:1000), and rabbit polyclonal phospho-Ser/Thr PKA substrate specific antibody (Cell Signaling, #9621, 1:1000). Secondary antibodies used include: pre-absorbed, polyclonal goat anti-rabbit IgG (H & L) HRP (Abcam, #ab97040, 1: 10000), and polyclonal goat anti-guinea pig IgG (H & L) HRP (Abcam, #ab97155, 1: 10000). Blots were used for reprobing with multiple antibodies were stripped (stripping buffer, consisting of (in mM): Tris HCl pH6.8: 62.5, 2-mercaptoethanol: 100, 2% SDS, 30 min at 50°C.). All immunoblot experiments were run in triplicate, representative images were shown on figures. For more details and for original blots see pages 17-23.

Immunprecipitation and *in vitro* phosphorylation of Ca_V1.2 protein

Immmunprecipitated Ca_V1.2 protein was used for *in vitro* phosphorylation studies. Rabbit polyclonal anti-Ca_V1.2 (Alomone, #ACC-003) antibody pre-incubated with Dynabeads Protein G (following the manufacturer Thermo Fisher Scientific instructions) used to pull out the Ca_V1.2 protein from tissue homogenates prepared from wt (N=5) and αMHC$^{403/+}$ hearts (N = 5). After protein concentration was measured, 2 Unit PKA catalytic subunit (Promega) was used per each μg of immunprecipitated protein to perform *in vitro* phosphorylation (2 hrs incubation at 37 °C in kinase buffer: 50 mM Tris-HCl, 9 mM $MgCl_2$, 0.5 mM ATP, pH 7.4, EDTA-free cOmplete mini (Roche), Phosphatase inhibitor cocktail IV and V (Merck Millipore). For detection of phosphorylation level of proteins phosphoprotein specific fluorescent ProQ Diamond Phosphoprotein Blot Stain (Molecular Probes) was used. After performing staining with. Pro-Q Diamond SyproRuby fluorescent stain was used to quantify total protein. After thorough washing

steps recommended by manufacturer, blots were blocked with 5% BSA in TBST and incubated with phospho-Ser/Thr PKA substrate specific primary antibody (Cell Signaling) overnight. Following signal detection (Luminata Forte, Western HRP substrate, Millipore), PVDF membrane was stripped (strip solution: 62.5 mM Tris-HCl pH 6.8, 100 mM β-mercaptoethanol, 2% SDS) and reprobed with anti $Ca_V1.2$ channel antibody (Alomone), then goat anti-rabbit HRP conjugate secondary antibody (Abcam). Densitometry was performed for each antibody specific bands using ImageJ software. Background subtracted intensity values were normalized to total protein fluorescent signal intensity, or $Ca_V1.2$ protein detected on the same blot

In vitro creatine kinase activity measurement

Creatine kinase activity measured (CLARIOstar microplate reader) on tissue homogenates prepared from wt and $\alpha MHC^{403/+}$ mice after *in vivo* ISO treatment, and from non-treated age-matched controls following the manufacturer's protocol (CK NAC-activated kit, Randox).

Immunocytochemistry

Precision cover glasses (thickness No. 1.5H, Marienfeld Superior) were sonicated for 20 mins in 1 M KOH to remove any fluorescent contaminants. Coverslips were then rinsed and subjected to several sonication steps in deionized water to release any KOH trapped between the coverslips. Washing/sonication continued until neutral pH was achieved indicating KOH was removed. Cleaned coverslips were kept in 70 % ethanol until required. On the day of isolation, ethanol was evaporated from single coverslips that were then coated with poly-L-lysine (0.01%; Millipore Sigma, St Louis, MO, USA) for 20 mins, rinsed with PBS, then coated with laminin (20 μg mL^{-1}; Life Technologies, Carlsbad, CA, USA). Coverslips remained in laminin for at least 45 mins before laminin was aspirated and freshly isolated wt and $\alpha MHC^{403/+}$ ventricular myocytes plated.
For confocal microscopy cells were fixed with 4% formaldehyde then solubilized using 0.5% TritonX-100. 5% BSA was used for blocking before the incubation with primary antibodies (as used for Western blot, 1:100 dilution, 2 hours at room temperature, then fluorescently labelled (Alexa Fluor 488 or Alexa Fluor 555) secondary antibodies (1:1000 dilution, 1 hour at room temperature). After mounting with ProLong™ Glass Antifade Mountant (ThermoFisher), cells were imaged with Nikon C2 confocal microscope coupled with NIS elements software.

For super-resolution studies, some coverslip adherent cells were treated for 10 mins with 100 nM isoproterenol (ISO; MilliporeSigma) prior to fixation in 100% ice-cold methanol (Fisher Scientific, Fair Lawn, NJ, USA) for 5 mins at -20°. Non-ISO treated controls remained bathed in PBS for 10 mins prior to fixation.

Fixed myocytes were thoroughly washed and blocked for 45 minutes at room temperature in 'blocking buffer' with the following composition: 20% SEA Block (Thermo Fisher Scientific, Rockford, IL, USA) and 0.05% v/v Triton X-100 (MilliporeSigma) in PBS. Adherent cardiac myocytes were incubated overnight at 4°C in blocking buffer containing mouse monoclonal anti-$Ca_V1.2$ (UC Davis/NIH NeuroMab Facility, UC Davis/NIH; clone N263/31; 1:100 dilution) with either rabbit polyclonal anti-caveolin-3 (Abcam, Eugene, OR, USA; ab2912; 1:1000 dilution) or rabbit polyclonal anti-β_1 adrenergic receptor (Abcam; ab3442; 1:1000 dilution). Cardiac myocytes were then thoroughly washed in PBS before undergoing a 1 h room temperature incubation in blocking buffer-diluted secondary antibodies including Alexa Fluor 647-conjugated goat anti-mouse IgG_{2b} and Alexa Fluor 555-conjugated goat anti-rabbit (Life Technologies; 1:1000 dilution). Imaging was performed following subsequent washes in PBS.

Super-resolution nanoscopy

Coverslips containing immuno-labelled cells were mounted onto glass depression slides (neoLab, Heidelberg, Germany) with a cysteamine (MEA)-catalase/glucose/glucose oxidase (GLOX) imaging buffer containing TN buffer (50 mmTris pH 8.0, 10 mm NaCl), a GLOX oxygen

scavenging system (0.56 mg mL^{-1} glucose oxidase, 34 µg mL^{-1} catalase, 10% w/v glucose) and 100 mm MEA. Twinsil dental glue (Picodent, Wipperfürth, Germany) and aluminum tape (T205-1.0 - AT205; Thorlabs Inc., Newton, NJ, USA) was used to seal the coverglass in place and to exclude oxygen (1). Cells were imaged on a super- resolution Ground State Depletion (GSD) microscope (Leica Microsystems, Wetzlar, Germany) in TIRF mode with 150 nm penetration depth as previously described (2-3). In brief, for each double-labelled cardiac myocyte, the Alexa Fluor 647-labelled $Ca_V1.2$ was imaged first using a 642 nm/500 mW laser to excite the dye. 60,000 frames were collected at 100 Hz via a Leica oil-immersion HC PL APO 160×/1.43 NA super-resolution objective. Next, a 532 nm/500 mW laser was used to excite the Alexa Fluor 555-labelled caveolin-3 or β_1 adrenergic receptor (60,000 frames collected). The collected frames were automatically reconstructed into super-resolution localization maps using Leica Application Suite (LAS AF) software. Fluorescence was detected through a Leica high-power TIRF quad filter cube (QGS HP-T) with emission band-pass filters of 421-477 nm, 497-519 nm, 547-621 nm, and 666-732 nm. Cluster area size was measured from binary masks of the localization maps with a 10 nm pixel size in ImageJ/Fiji as previously described (4). ImageJ/Fiji plug-in, JACoP, was used to determine the shortest intermolecular distances between Cav3 and $Ca_V1.2$, as well as β_1 adrenergic receptor (β_1AR) and $Ca_V1.2$. Output data was used to generate intermolecular distance histograms which were then fitted in GraphPad Prism software (GraphPad, San Diego, CA, USA) with a sum of two Gaussian functions based on previously described methods (5)

Generation of patient-specific human induced pluripotent stem cells (hiPSCs) and general cell culture maintenance

Human induced pluripotent stem cells were derived from a patient carrying the FHC causing mutation p.R403Q in *MYH7* (48). Peripheral blood mononuclear cells (PBMCs) were isolated and reprogrammed into hiPSCs as previously described (48). iPSC colonies were maintained in defined, feeder cell-free medium, mTeSR1 PLUS™ (StemCell Technologies, Tullamarine, AUS) and the extracellular matrix Matrigel hESC-qualified matrix (Corning Inc, NY, USA), and passaged as aggregates using Gentle Cell Dissociation Reagent (StemCell Technologies). CRISPR-Cas9 gene editing was utilised to correct the R403Q variant in patient-derived cell lines. See Online Supplementary Material for expanded Methods including differentiation protocol.

CRISPR-Cas9 gene editing of hiPSCs

CRISPR-Cas9 gene editing (8) was utilised to correct the R403Q variant in patient-derived cell lines. Guide RNAs were designed using an online tool (crispr.mit.edu) and ordered as oligos to be cloned into pSpCas9(BB)-2A-Puro (PX459) V2.0 which was a gift from Feng Zhang (Addgene plasmid #62988, RID:Addgene_62988). Homology directed repair (HDR) donor oligos were designed to flank the Cas9 cut site and ordered as Ultramer DNA oligonucleotides that contained 2 phosphorothioate bonds on each end of the oligo (Integrated DNA Technologies, IA, United States). hiPSCs were plated as single cells and transfected 24 h later with 1 µg plasmid and 5 µL 100µM donor oligo using Lipofectamine™ Stem (Thermo Fisher, MA, United States) according to manufacturer's protocol. 24 h after transfection, cells were selected with 0.5 µg/mL puromycin for a further 24 h. Single cells were selected into a 96-well plate for Sanger sequencing to determine successfully edited clones which were further expanded into established cell lines. Off target analysis involved Sanger sequencing of ten potential guide RNA off-target sites (http://www.rgenome.net/cas-offinder/), Sanger sequencing of all TP53 exons and molecular karyotyping (Victorian Clinical Genetics Services, VIC, AUS) to ensure genomic integrity.

	Sequence 5' to 3'
Guide RNA for correcting *MYH7* Arg403Gln	CATTGCCCACTTTCACCTGA

Donor template for correcting *MYH7* Arg403Gln	C*C*TCATGGGGCTGAACTCAGCCGACCTGCTCAAGGGGC TGTGCCATCCTCGGGTGAAAGTGGGCAATGAGTACGTCA CCAAGGGGCAGAATGTCCAGC*A*G

Differentiation of hiPSC-CMs

hiPSC cultures at 70-80% confluence were dissociated into a single cell suspension with TryPLE for 7 minutes at 37˚C, 5% CO_2. Cells were then plated between 450,000 – 500,000 cells per well of a Matrigel coated 12-well tissue culture plate in mTeSR1 PLUS™ (StemCell Technologies) supplemented with 10 μM ROCK inhibitor (Y-27632) (Reprocell, Beltsville, USA). After 24 hrs, media was replaced with mTeSR1 PLUS™ (StemCell Technologies). hiPSCs were cultured for 2-3 days, until >95% confluency was achieved, hiPSCs are differentiated into cardiomyocytes using the StemDiff Cardiomyocyte differentiation kit (StemCell Technologies) as per manufacturers protocol. Beating hiPSC-CMs were observed at 10-12 days post-differentiation.
At day 15 (± 2 days) post-differentiation hiPSC-CMs were dissociated using a two-step protocol described previously (9). First, hiPSC-CMs were incubated with 0.2% Collagenase Type I (Thermo Fisher), in phosphate buffered saline (PBS) supplemented with 20% fetal bovine serum (FBS), for 45 minutes at 37 °C, 5% CO_2 to break up the extracellular matrix. Following centrifugation at 300 × *g* for 3 minutes, hiPSC-CMs were incubated in 0.25% Trypsin with EDTA (Thermo Fisher) for 10 minutes at room temperature. After trypsin neutralisation, hiPSC-CMs were filtered through a 100 μm cell strainer (pre-wet with PBS), centrifuged for 300 × *g* for 3 minutes, then resuspended at the required density for plating in control media α-MEM GlutaMAX, 10% FBS, 200 μM L-ascorbic acid 2 phosphate sesquimagnesium salt hydrate, and 1% Penicillin/Streptomycin and seeded as single cells into a Matrigel-coated KIC plate. Media was changed 2 days after seeding to control media without FBS and subsequently maintained for 15 (± 2 days) days prior to voltage measurements.

Immunostaining of iPSC-CMs

At day 36 post differentiation cells were washed with PBS, fixed in 4% paraformaldehyde for 15 min, permeabilised with 1% Saponin for 15 mins and then blocked in 3% BSA for 30 min at room temperature. Cells were then incubated with 1:1000 mouse-anti TNNT2 primary antibody (Invitrogen, Massachusetts, USA) for 3 h at room temperature in 3% BSA. After washing 3 times with PBS for 5 min, cells were incubated in 1:250 Alexa Fluor 488 Goat anti-mouse IgG secondary antibody (Thermo Fisher) for 1 h at room temperature in 3% BSA. Cells were washed 3 times in PBS for 5 min and NucBlue™ (Thermo Fisher) was added for the last wash. Coverslips were mounted with ProLong™ Gold Antifade Mountant (Thermo Fisher) and imaged on the Leica SP8 confocal microscope (Leica, Wetzlar, DE).

Kinetic imaging cytometry (KIC) in iPSC-CMs

Voltage measurements were performed with the kinetic imaging cytometry (KIC) platform (Vala Sciences, San Diego, USA). Patient derived R403Q (+/-) and CRISPR corrected isogenic control hiPSC-CMs were dissociated and seeded on Matrigel coated 96-well assay plates (CELL STAR μclear, Greiner Bio-One GmbH, Frickenhausen, DEU). After 15 (± 2 days) incubation, hiPSC-CMs in each well of the assay plate were loaded with 5 μg/mL of Hoescht nuclear stain (Thermo Fisher Scientific) and half the recommended concentration of FluoVolt listed in the manufacturers protocol, diluted in phenol-free RPMI 15640 media (Thermo Fisher Scientific) supplemented with 1 mM Ca^{2+}. Cells were incubated for 20 mins, and replenished with fresh phenol-free RPMI 15640 media supplemented with 1 mM Ca^{2+} before fluorescent images were acquired using an IC200-KIC™ with CyteSeer scanner v2.2.32.0 software (Vala Sciences, San Diego, USA). All incubation steps, as well as the recordings, were performed with environmental control at 37 °C and 5% CO_2.

For each well of the 96-well plate, a single fluorescent image was first acquired by excitation of the Hoescht nuclear indicator using a standard DAPI filter (excitation at 350 λ, emission at 470 λ). A kinetic series of high content fluorescent images were then subsequently acquired at a sampling frequency of 1000 Hz, using a standard FITC filter to excite the FluoVolt membrane voltage indicator (excitation at 490 λ, emission at 525 λ). hiPSC-CMs were stimulated, but not recorded for 10 seconds, followed by 20 seconds of recording where there was 10 seconds of DC stimulation at 0.5Hz with 10 ms pulses, for a total of 5 pulses. Another 10 seconds of spontaneous activity was recorded immediately after the stimulation.
A final concentration of 1 μM ISO (Sigma) diluted in phenol-free RPMI media supplemented with 1 mM Ca^{2+} was delivered using an automated robotic platform (Vala Sciences) in order to maintain environmental control. The previous acquisition protocol was repeated following a 2 minute incubation. Images were stored for offline analysis using the CyteSeer v2.8.0.91 software (Vala Sciences). From each well of a 96-well plate, the single nuclear fluorescent image from each condition (i.e. before and after ISO addition) underwent segmentation processing to identify individual hiPSC-CM nuclei within the field of view. The 1998 kinetic images per conditions were then processed to identify the cell boundary for each nuclei. Using this segmentation mask, fluorescent signals from each individual hiPSC-CM could then be sampled to create a surrogate hiPSC-CM voltage signal over time. Individual parameters/measures of the voltage action potential duration are shown in the relevant figures and figure legends.

Statistical analysis

Data analysis, plotting and statistical tests were performed using Matlab (Mathworks, MA, USA), Microsoft Excel (Microsoft Office 2016, Microsoft) and GraphPad Prism v8.4.3 (GraphPad Software, San Diego, USA). All data are expressed in mean ± SEM. The number of experimental data points (n) for each experimental condition is given in the text, figure or legend. N= number of animals used. The Shapiro-Wilk normality test was used to assess whether the data were normally distributed. If the data were normally distributed a Brown-Forsyth and Welch ANOVA was used to analyse differences between wt and αMHC403$^{/+}$ groups. Where data were not normally distributed a Kruskal-Wallis ANOVA was performed. A Dunn's test was used to correct for multiple comparisons. p-values <0.05 were considered significant.

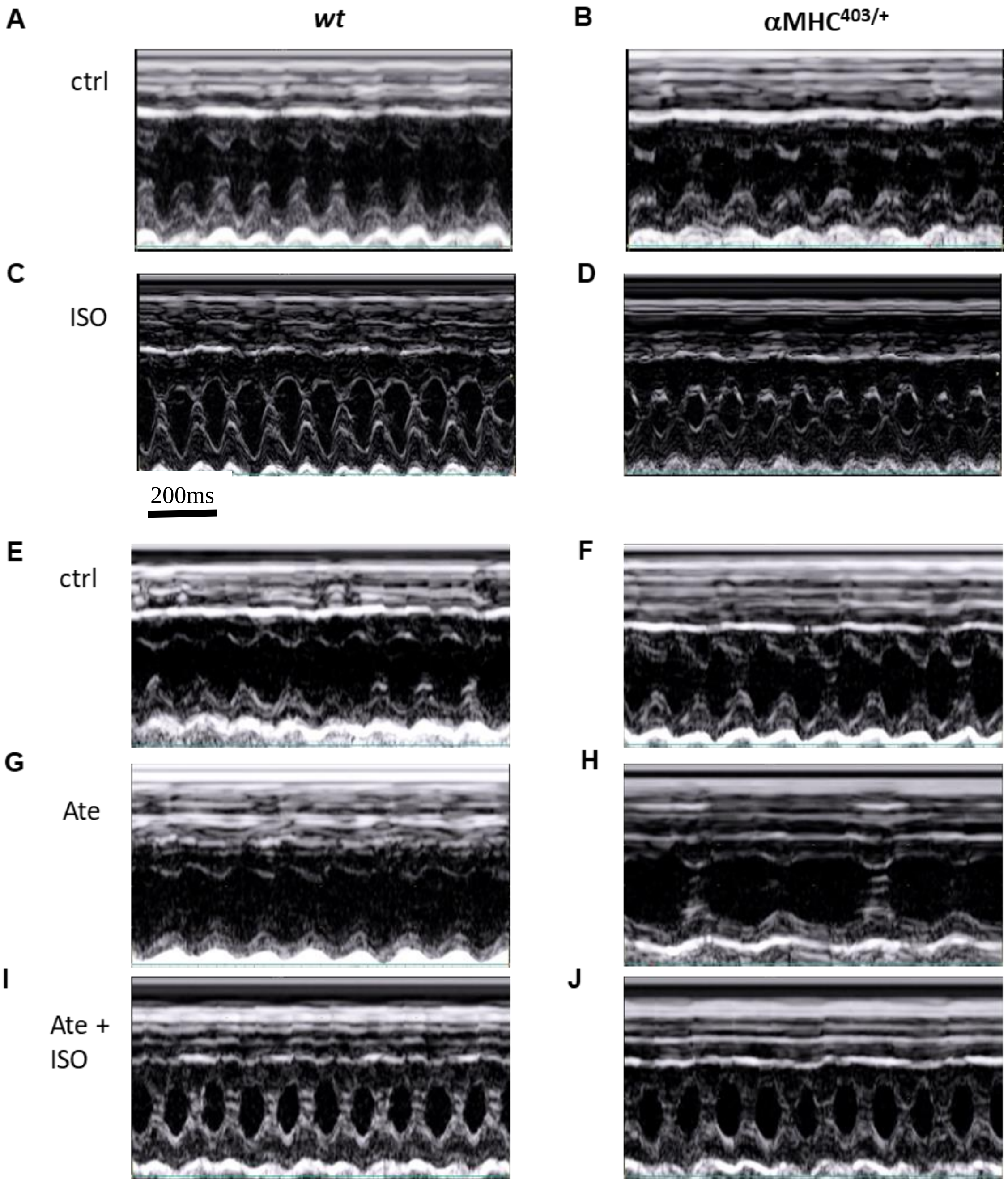

Fig. S1. **Echocardiography ultrasound studies performed on wt (left column) and αMHC403/+ mice (right column).**

Images acquired prior to (A-B) and immediately following (C-D) i.p. injection of 20 mg/kg ISO under light methoxyflurane anesthesia. Further ultrasound studies were performed following i.p injection of the β-blocker atenolol (Ate) in wt and αMHC403/+ mice. Representative echocardiograms before (E-F) and after 1 mg/kg atenolol (G-H), followed by 20 mg/kg ISO (I-J) demonstrating an increase in the heart rate in the presence of atenolol.

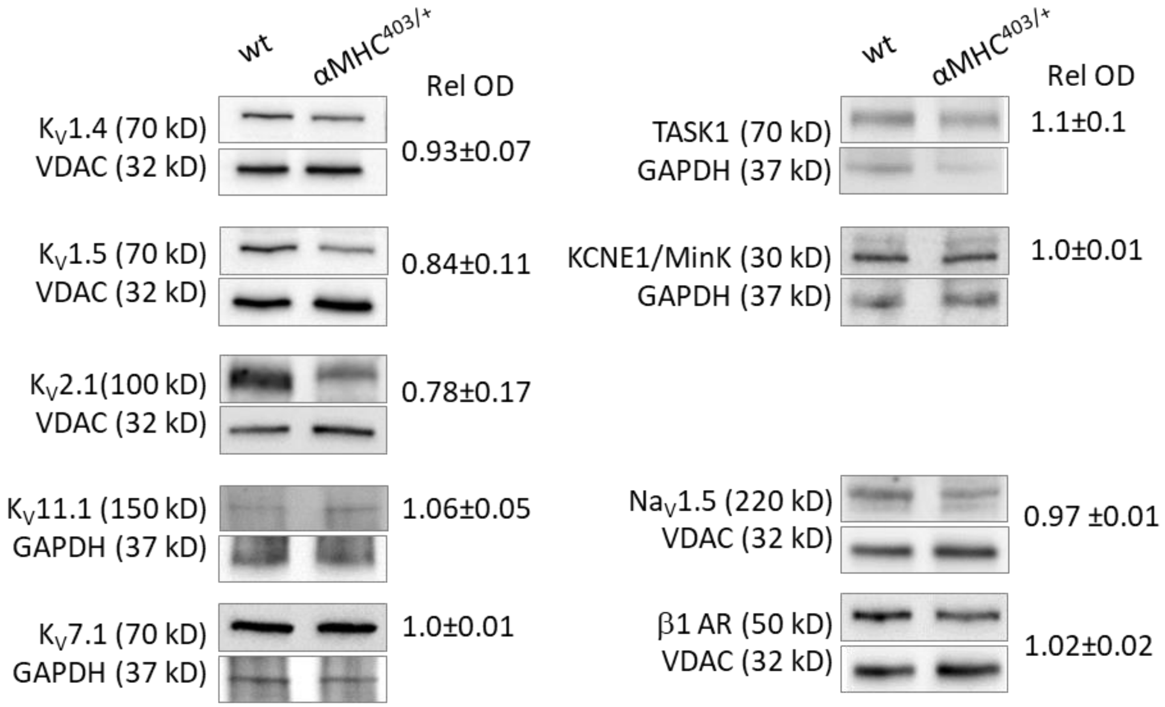

Fig. S2. **Representative Western blot images for K_v potassium channel isoforms and auxiliary subunits as indicated, Na_v1.5 sodium channel and β1 adrenergic receptor (AR) proteins**.

There was no significant difference in the expression level between wt and αMHC403/+ hearts for proteins as shown, detected on blots with total heart homogenates from four wt and four αMHC403/+ hearts and repeated in triplicate. Relative optical density values calculated using VDAC or GAPDH as loading controls as indicated.

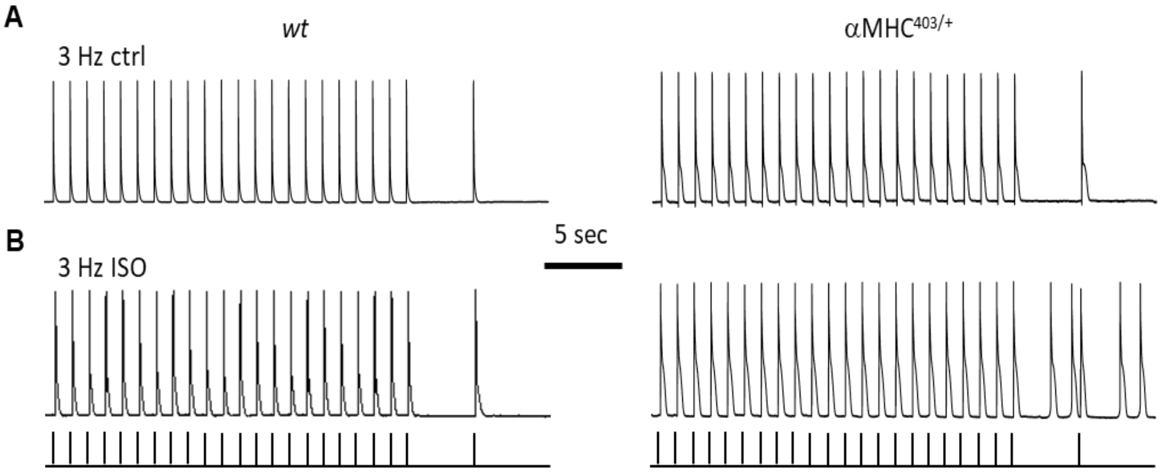

Fig. S3. Representative action potential train recordings from ventricular myocytes isolated from wt (left column) and αMHC403$^{/+}$ mice (right column). Cells were paced at 3 Hz, 0.2 ms, suprathreshold stimulus in current clamp mode under control conditions (A) or the presence of 100 nM ISO (B).

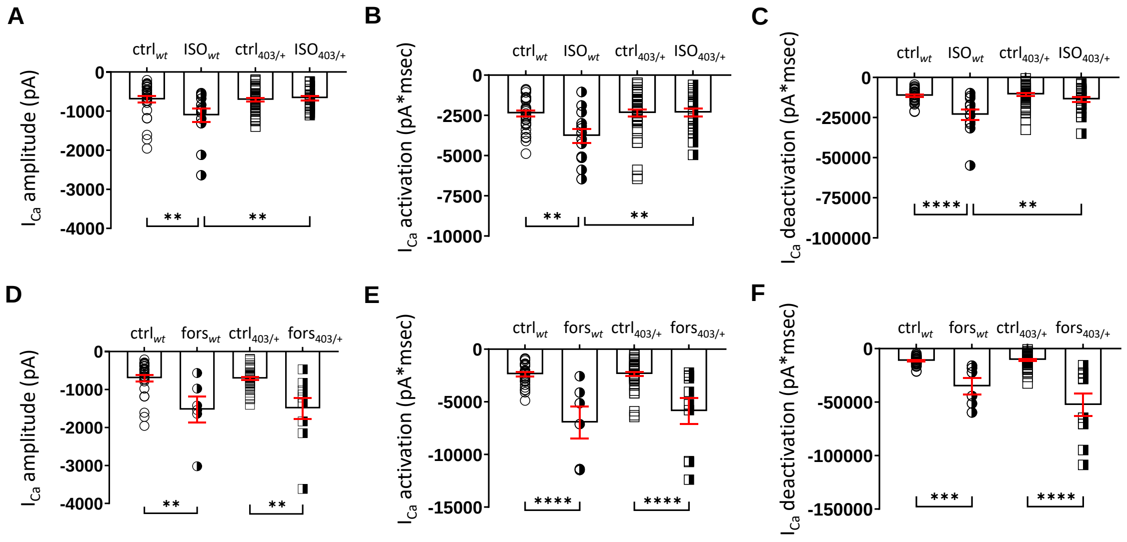

Figure S4. Activation and deactivation parameters of I_{Ca} currents.
Currents were measured under control conditions or in the presence of 100 nM ISO in wt and αMHC403$^{/+}$ ventricular myocytes. *p < 0.05, number of asterisks representing increased significance with p value

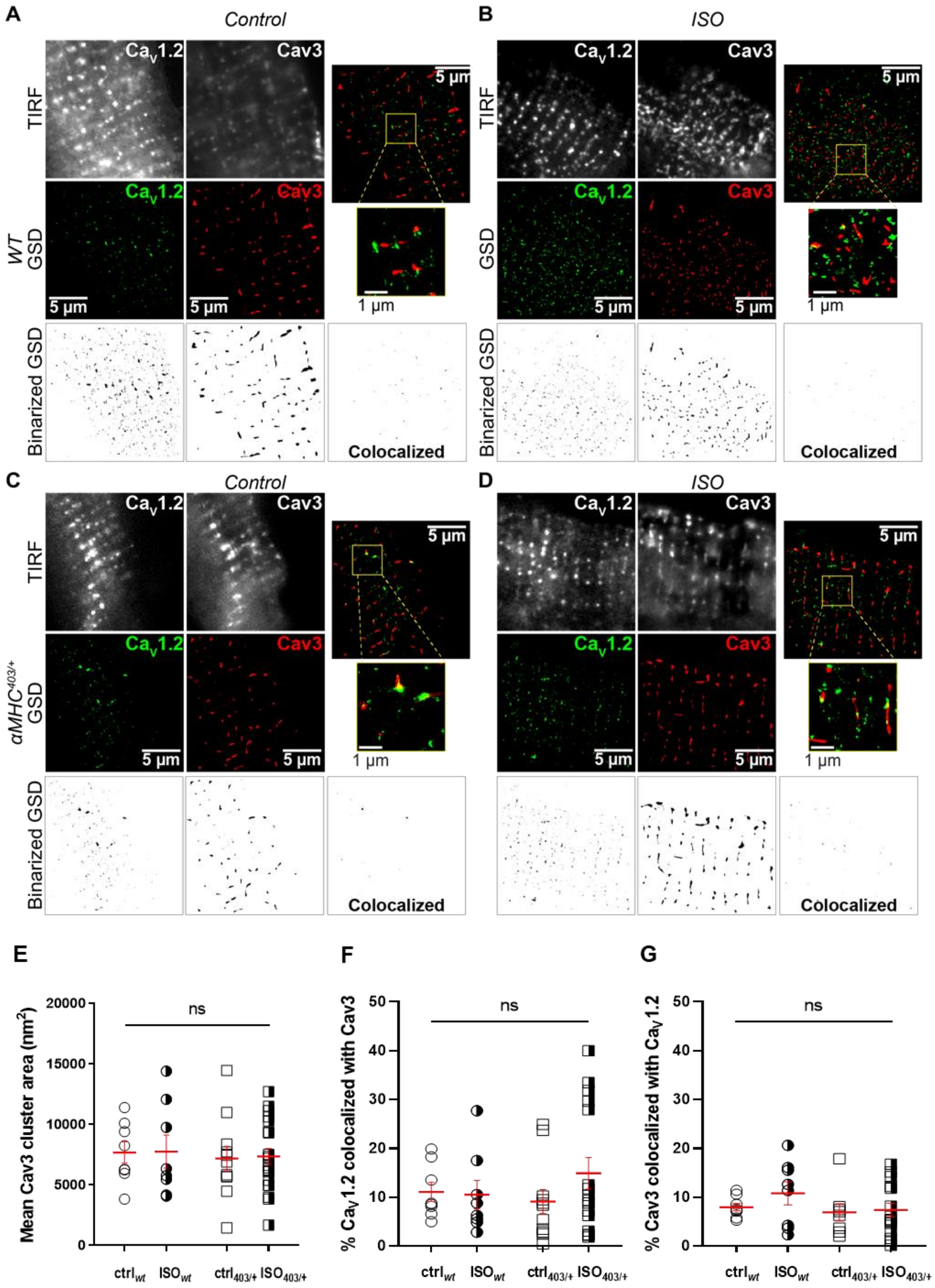
A
Control
B
ISO
$Ca_V1.2$
Cav3
TIRF
WT GSD
5 µm
1 µm
Binarized GSD
Colocalized
C
D
αMHC403/+ GSD
E
F
G
ns
Mean Cav3 cluster area (nm²)
% $Ca_V1.2$ colocalized with Cav3
% Cav3 colocalized with $Ca_V1.2$
ctrlwt
ISOwt
ctrl403/+
ISO403/+

Fig. S5. Colocalization of Caveolin 3 (Cav3) with $Ca_V1.2$ is unaltered in αMHC403$^{/+}$ myocytes.

(A-B) TIRF images (top row), super-resolution GSD localization maps (middle row) and binarized images (bottom row) of immunostained $Ca_V1.2$ channels and Cav3 in representative fixed adult ventricular myocytes isolated from wt mice under control (A) or ISO-stimulated conditions (B). Merged two-channel image showing relative distributions of $Ca_V1.2$ and Cav3 is shown in the third column with the colocalized binarized image. (C-D) Same layout format for myocytes isolated from αMHC403$^{/+}$ mice. E: Mean Cav3 cluster areas ± S.E.M. (indicated by red lines and error bars) are summarized for each condition in the aligned dot plot. (F) % colocalization of $Ca_V1.2$ with Cav3. (G) % colocalization of Cav3 and $Ca_V1.2$. n = 7-18 cardiac myocytes, from N = 3-7 mice.

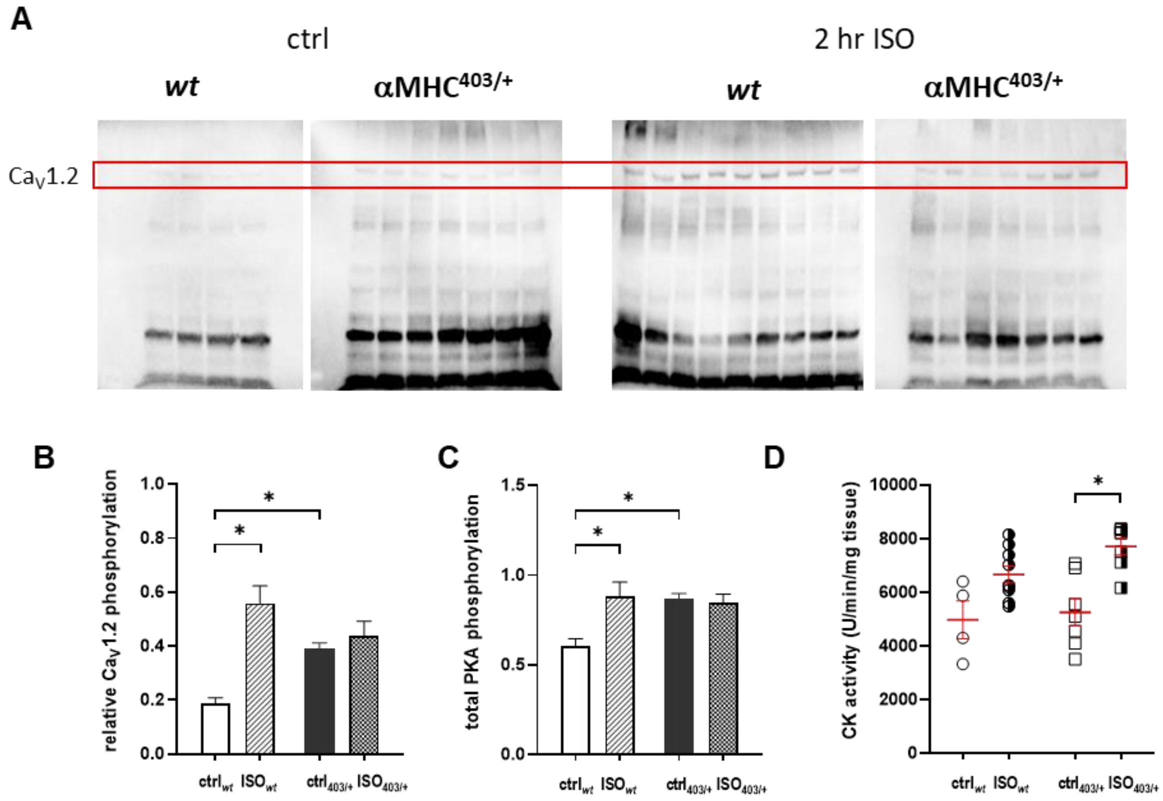

Fig. S6. Ca$_V$1.2 is phosphorylated in hearts of αMHC403$^{/+}$ mice.
Representative western blots (A) and quantitated relative Ca$_V$1.2 phosphorylated protein (B) and total phosphorylated protein (C) of heart tissue samples snap frozen after 2 hours ISO treatment (N=7 hearts) compared with non-treated age-matched controls (ctrl; N=9 hearts) as indicated. (D) Creatine kinase activity measured on the same heart tissue samples following exposure to ISO as indicated (CK NAC-activated kit, Randox). *p < 0.05 as indicated.

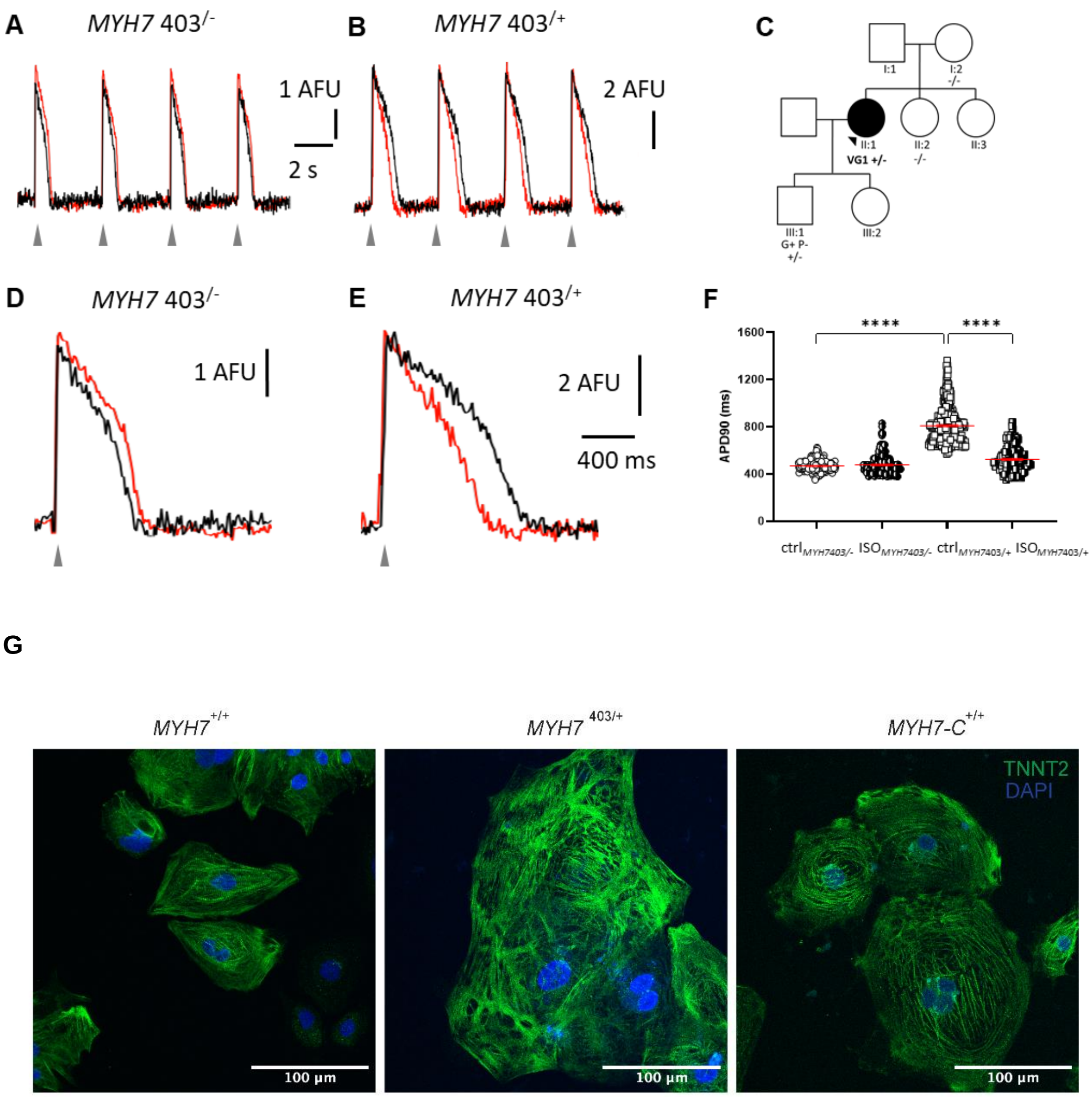

Fig. S7. Effect of ISO on action potentials from hiPSC-CM's from a patient carrying the *MYH7* mutation.
Representative action potentials recorded at 0.5Hz from hiPSC-CMs from a patient carrying the *MYH7* mutation (*MYH7 403/+* panel B and further enlarged in panel E) and isogenic CRISPR corrected hiPSC-CM control (*MYH7 403/-* panel A and further enlarged in panel D). Patient carrying the *MYH7* mutation indicated as filled symbol on family pedigree (C). hiPSC-CMs identified as *MYH7* 403$^{/+}$ had prolonged action potentials. Shown in the absence (black trace) or presence of 1 µM ISO (red trace). (F) Action potential durations at 90% repolarization for *MYH7* 403$^{/-}$ (n = 192 and 204 cells from three batches of differentiation) and *MYH7* 403$^{/+}$ (n = 406 and

221 cells from six batches of differentiation; (G) Representative Day 36 differentiated iPSCs immunostained with TNNT2 and DAPI. *MYH7*$^{+/+}$: unaffected sibling, *MYH7*$^{403/+}$: mutant cell, *MYH7*-C$^{+/+}$: CRISPR corrected isogenic control. *$p < 0.05$, number of asterisks representing increased significance with p value) in the absence (ctrl) or presence of isoproterenol (ISO).

SI References

1. Nahidiazar L, Agronskaia AV, Broertjes J, van den Broek B and Jalink K. Optimizing imaging conditions for demanding multi-color super resolution localization microscopy. PLoS One. 2016;11:e0158884.
2. Ito DW, Hannigan KI, Ghosh D, Xu B, Del Villar SG, Xiang YK, Dickson EJ, Navedo MF and Dixon RE. beta-adrenergic-mediated dynamic augmentation of sarcolemmal $Ca_V1.2$ clustering and co-operativity in ventricular myocytes. J Physiol. 2019;597:2139-2162.
3. Dixon RE, Moreno CM, Yuan C, Opitz-Araya X, Binder MD, Navedo MF and Santana LF. Graded Ca^{2+}/calmodulin-dependent coupling of voltage-gated $Ca_V1.2$ channels. Elife. 2015;4.
4. Dixon RE, Vivas O, Hannigan KI and Dickson EJ. Ground state depletion super-resolution imaging in mammalian cells. J Vis Exp. 2017.
5. Prada MP, Syed AU, Buonarati OR, Reddy GR, Nystoriak MA, Ghosh D, Simo S, Sato D, Sasse KC, Ward SM, Santana LF, Xiang YK, Hell JW, Nieves-Cintron M and Navedo MF. A Gs-coupled purinergic receptor boosts Ca^{2+} influx and vascular contractility during diabetic hyperglycemia. Elife. 2019;8.
6. Viola H, Johnstone V, Cserne Szappanos H, Richman T, Tsoutsman T, Filipovska A, Semsarian C and Hool L. The L-type Ca^{2+} channel facilitates abnormal metabolic activity in the cTnI-G203S mouse model of hypertrophic cardiomyopathy. J Physiol. 2016;594:4051-70.
7. Viola HM, Shah AA, Johnstone VPA, Cserne Szappanos H, Hodson MP and Hool LC. Characterization and validation of a preventative therapy for hypertrophic cardiomyopathy in a murine model of the disease. Proc Natl Acad Sci U S A. 2020;117:23113-23124.
8. Ran FA, Hsu PD, Wright J, Agarwala V, Scott DA and Zhang F. Genome engineering using the CRISPR-Cas9 system. Nat Protoc. 2013;8:2281-2308.
9. Mills RJ, Titmarsh DM, Koenig X, Parker BL, Ryall JG, Quaife-Ryan GA, Voges HK, Hodson MP, Ferguson C, Drowley L, Plowright AT, Needham EJ, Wang QD, Gregorevic P, Xin M, Thomas WG, Parton RG, Nielsen LK, Launikonis BS, James DE, Elliott DA, Porrello ER and Hudson JE. Functional screening in human cardiac organoids reveals a metabolic mechanism for cardiomyocyte cell cycle arrest. Proc Natl Acad Sci U S A. 2017;114:E8372-E8381.

Original, full representative Western blot images

Full, unedited representative Western blots for Figure 3G

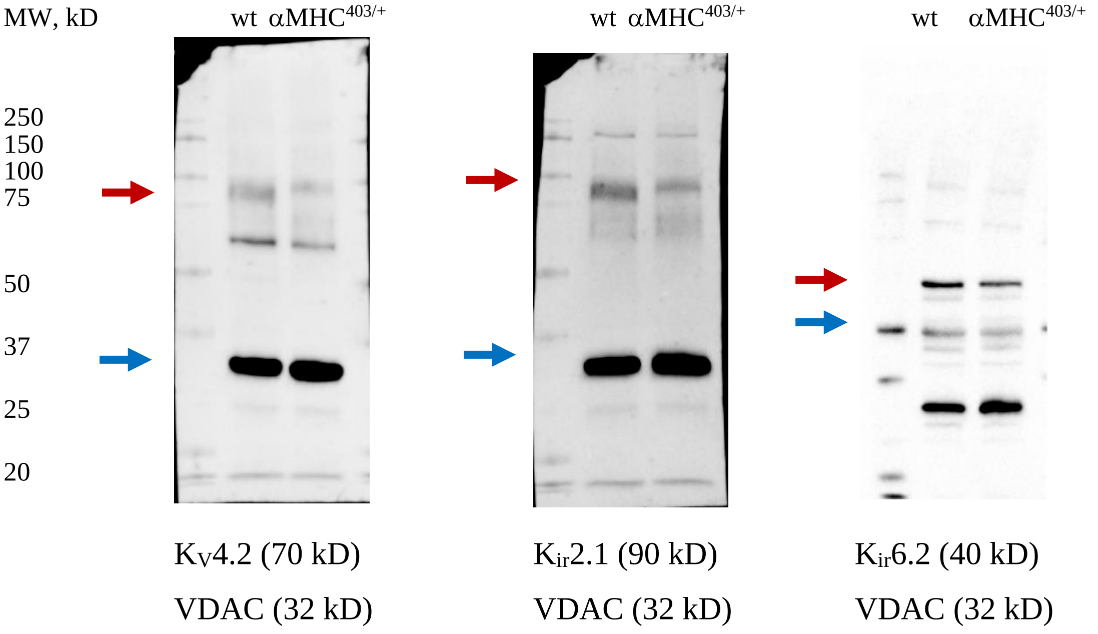

Full unedited representative Western blots for Figure 4

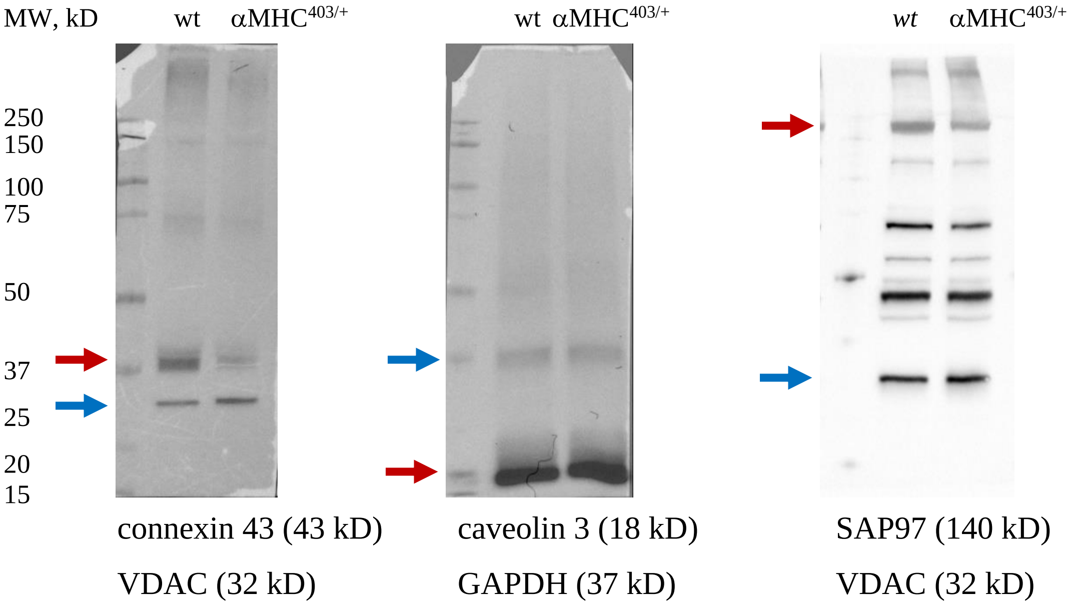

Ref: Submission ID 896f7808-7e8e-4dbf-b775-1894650fd0f5 R1

Full unedited representative Western blots for Figure 5F

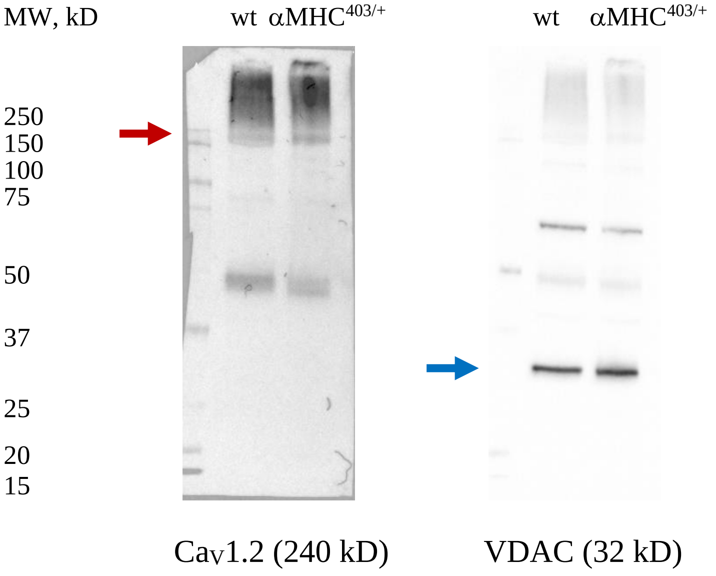

Ref: Submission ID 896f7808-7e8e-4dbf-b775-1894650fd0f5 R1

Full unedited representative Western blots for Figure 5G

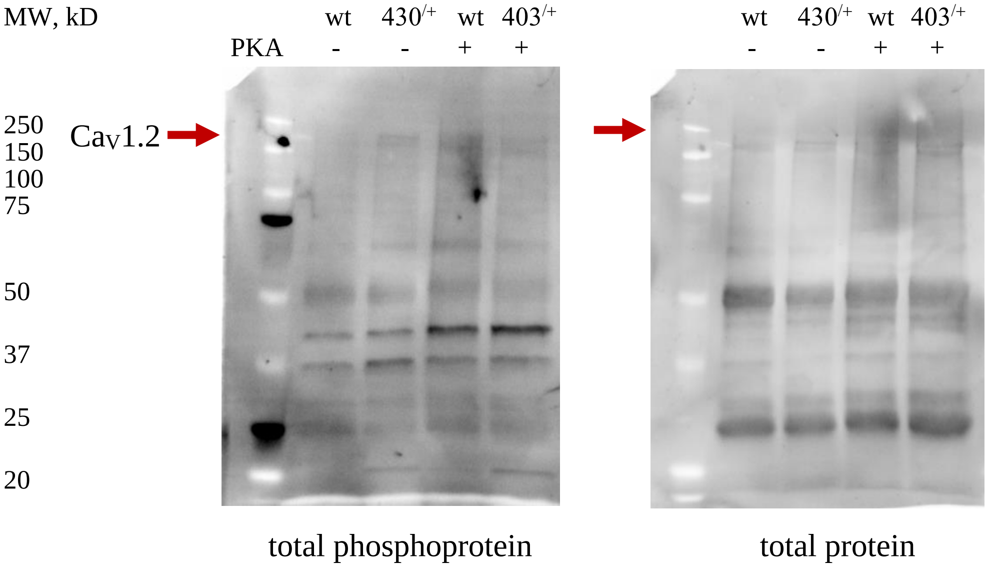

Western blot replicates used for densitometry analysis

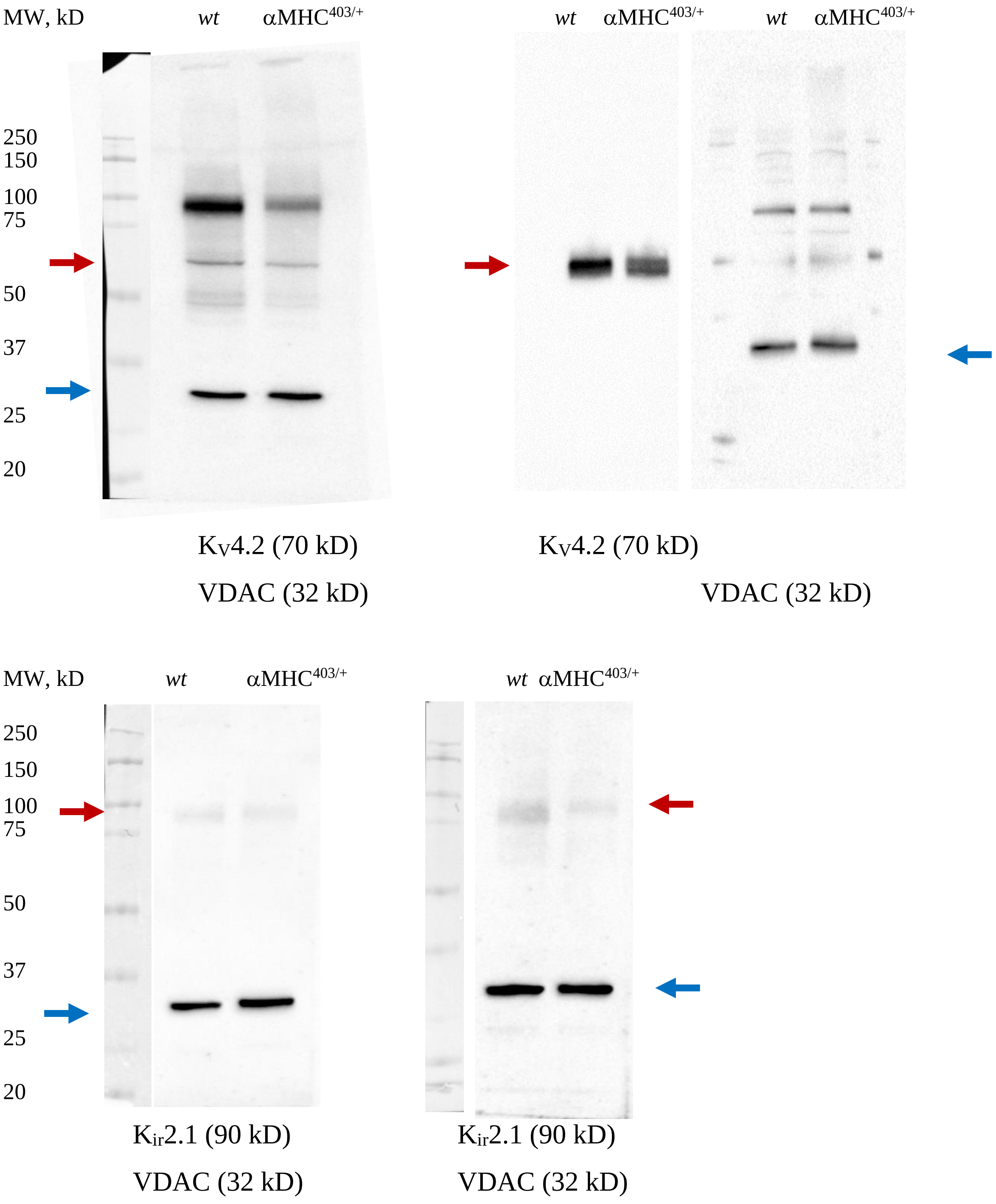

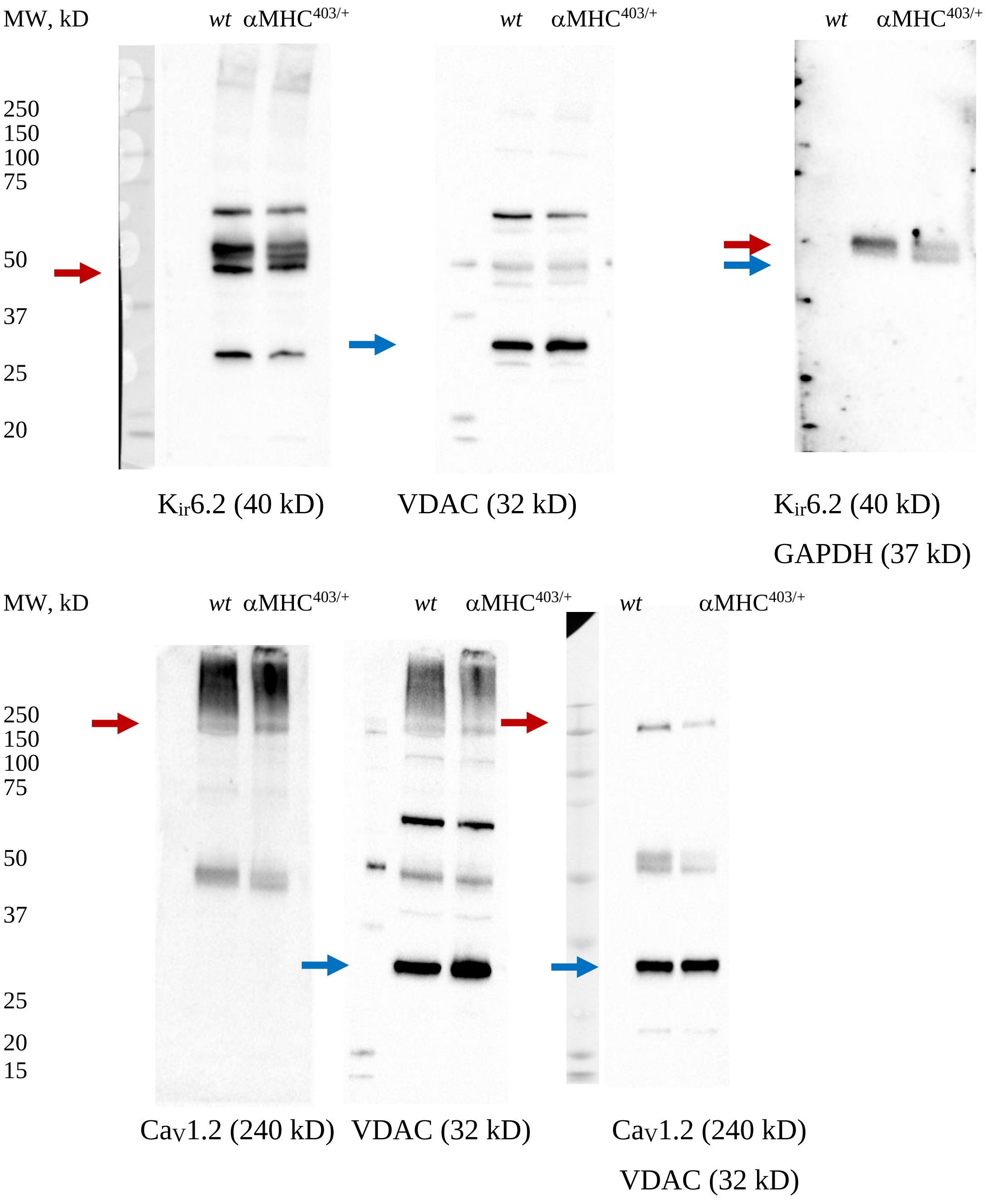
MW, kD
wt αMHC403/+
wt αMHC403/+
wt αMHC403/+
250
150
100
75
50
37
25
20
Kir6.2 (40 kD)
VDAC (32 kD)
Kir6.2 (40 kD)
GAPDH (37 kD)
MW, kD
wt αMHC403/+
wt αMHC403/+
wt αMHC403/+
250
150
100
75
50
37
25
20
15
Cav1.2 (240 kD)
VDAC (32 kD)
Cav1.2 (240 kD)
VDAC (32 kD)

Ref: Submission ID 896f7808-7e8e-4dbf-b775-1894650fd0f5 R1

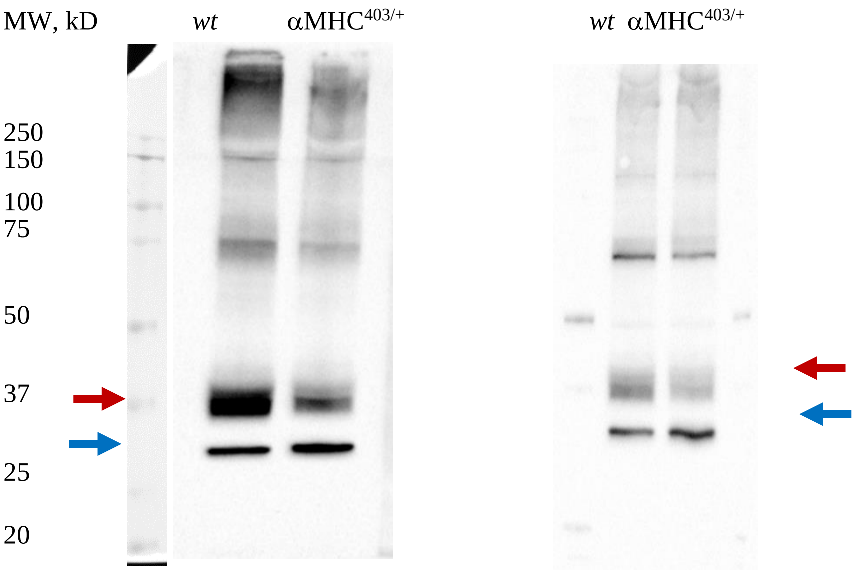

connexin 43 (43 kD) connexin 43 (43 kD)

VDAC (32 kD) VDAC (32 kD)

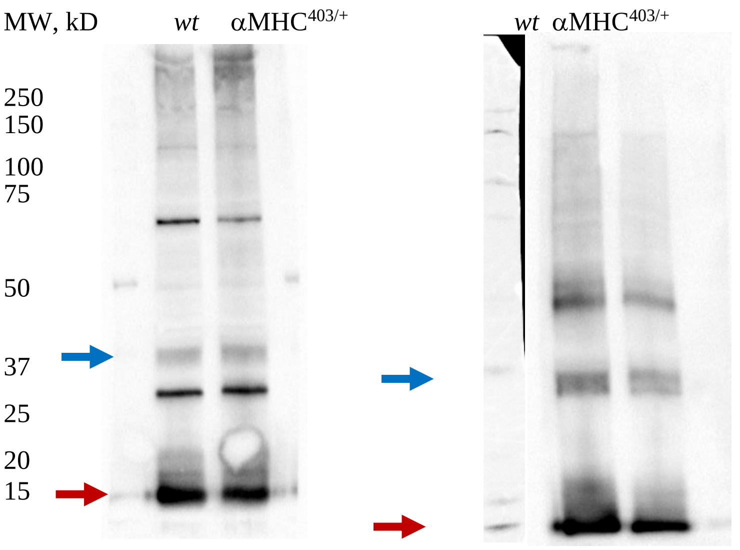

caveolin 3 (18 kD) caveolin 3 (18 kD)

GAPDH (37 kD) GAPDH (37 kD)

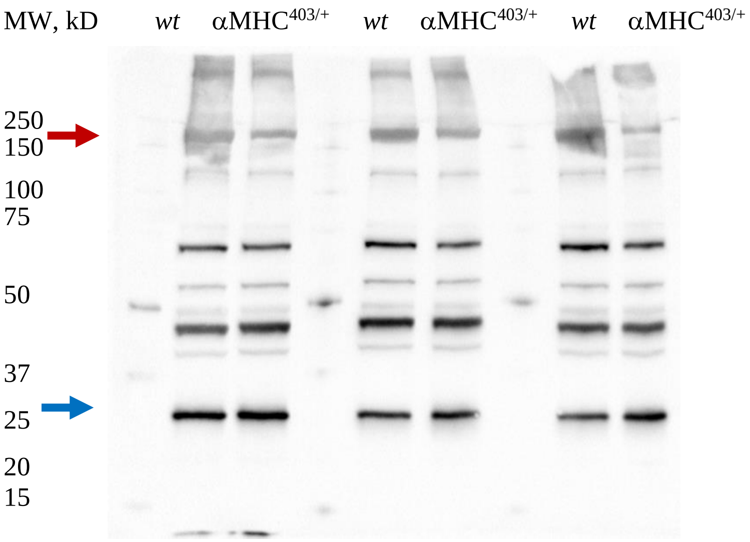

SAP97 (140 kD) SAP97 (140 kD) SAP97 (140 kD)

VDAC (32 kD) VDAC (32 kD) VDAC (32 kD)

Monitoring Cover Sheet

1) CONTACT DETAILS

**AEC Protocol #**	**RA/3/100/1617**	
**Protocol Title :**	Prevention of hypertrophy and arrhythmias in mouse models of cardiomyopathy	
**Monitoring Start Date :**		
**Chief Investigator :**	Professor Livia Hool	**Ph:** [illegible]
**Emergency Contact :**	Professor Livia Hool	**Ph:** [illegible]
**Monitor 1 :**	Dr Henrietta Cserne Szappanos	**Ph:** [illegible]
**Monitor 2 :**	Ms Teagan Er	**Ph:** [illegible]
**Monitor 3:**	Ms Khanh Vu<br>✓ under supervision until competent	**Ph:** [illegible]
**Monitor 4:**		
**Monitor 5:**		
**Person responsible for euthanasia :**	Professor Livia Hool	**Ph:** [illegible]
**Other experts :**		**Ph:**
**Animal Welfare Officer**	**AWO**	Ph: [illegible]

2) SPECIES / PHENOTYPE / MODEL ISSUES

Mus musculus / cTnI-G203S and αMHC$^{403/+}$ mutant models of familial hypertrophic cardiomyopathy

The αMHC$^{403/+}$ (403) mice demonstrate a normal life span with no difference in survival at 52 weeks compared to wt age-matched controls.

3) MONITORING CRITERIA AND SCORING

**Standard AEC recommended criteria**	**0**	**1**	**2**
Activity – i.e. movement around the cage Bright, Alert, Responsive (BAR)	Normal – mobile and active	Somewhat and/or intermittent stillness as compared to others	Will only move if approached or reluctant to move when touched. Moderately reduced activity, dull, lethargic
Body Posture	Normal	Somewhat and/or intermittent hunched appearance	Moderate/continuous hunching and still
Social Behaviour	Normal	Somewhat or intermittently separate from others	Completely separate or isolated from others
Coat quality/skin condition	Normal. Coat flat and glossy, showing evidence of normal grooming behaviour.	Somewhat ruffled or unkempt or mild piloerection. Somewhat scaly, scurfy or reddened or discolouration of skin.	Moderately ruffled or moderate piloerection consistent with failure to groom. Moderate scale and scurf, scratches or moderate discolouration of skin.

© The University of Western Australia 2015 CRICOS Provider Code: 00126G

Body Condition	Animal is well-conditioned. Vertebrae and dorsal pelvis not prominent; palpable with slight pressure.	NA	Animal is under conditioned. Segmentation of vertebral column evident. Dorsal pelvic bones readily palpable.
Facial Expression	Normal	Slight or intermittent narrowing of the eyes and/or flattening of the ears.	Moderate narrowing of the eyes and/or flattening of the ears.
**Project specific criteria**	**0**	**1**	**2**
Injection site	Normal	Slight swelling and/or redness at wound edges	Moderate swelling and/or redness at wound edges/wound discharge
**Other presenting signs/symptoms** **Although the most relevant monitoring criteria have been selected, if a circumstance arises where there are additional presenting signs or behaviours, these must be acknowledged, scored, recorded and reported to the AWO as an adverse event.**	Slight or intermittent or possible deviation from normal for this sign.	Moderate or consistent or definite deviation from normal but not marked, for this sign.	

4) MONITORING FREQUENCY

Describe monitoring regime including frequency of animal assessment and weighing. Select frequency that will adequately identify potential issues (including those described in section17D).

The mice will be monitored twice a week, and weighed weekly in PCF or M Block. Mice undergoing echocardiography or electrocardiography assessment will be monitored during procedures, at the end of the day, and the following day. The mice will also be monitored during and after injection. The mice will be checked again at the end of the day, and the following day.

**Type of recording sheet** Insert **X** *to indicate type of recording sheet/s attached)*			
General **[X]**	Anaesthesia **[ ]**	Post Procedure **[X]**	Other **[X]** Specify: **Anaesthesia - Short gaseous for restraint**

5) ACTIONS AND INTERVENTIONS

**Total Welfare Impact Score** *Add together all individual monitoring criteria scores for a Total Welfare Impact Score.*	**Actions/Interventions**
**0**	• No interventions required
**1**	• Monitor once daily<br>• Consider analgesia. (As described in the approval or following veterinary authorisation)<br>• *For this project, select appropriate actions and interventions to minimise impact on animal welfare for this total score.*
**2 - 4**	• Monitor twice daily<br>• Weigh

© The University of Western Australia 2015 CRICOS Provider Code: 00126G

	• Consider analgesia. (As described in the approval or following veterinary authorisation).<br>• Assess for euthanasia<br>• *For this project, select appropriate actions and interventions to minimise impact on animal welfare for this total score.*
**5**<br>**Humane end-point**<br>Point when animals should be humanely killed *(regardless of whether the study aims have been achieved)*	• Immediate euthanasia<br>• Complete reporting documentation and submit to facilities staff/manager and AWO if required.
**Additional Specific Interventions**<br>*(i.e. to manage project specific criteria indicated above, or specific husbandry care)*	*For this project, select appropriate actions and interventions to minimise impact on animal welfare for this specific health condition.*

6) AEC INTERVENTIONS for Body Weight Loss and Subcutaneous Tumour Size *(as appropriate to the project)*

Please refer to *Guidelines on the Induction of Tumours and Monitoring of Animal Welfare* and *Guidelines on Body Weight Deficit and Monitoring of Animal Welfare* documents at: www.research.uwa.edu.au/staff/forms/animals			
NB. When body weight loss Threshold 2 is set at 10% the AEC does not require a dual threshold. Therefore Threshold 1 should read N/A			
**Weight loss %***	**Threshold 2:**	**10%**	• If the body weight deficit in any animal reaches this approved second threshold euthanasia must be performed.<br>• Complete reporting documentation and submit to facilities staff/manager and AWO if required.

7) INSTRUCTIONS for the conduct of the monitoring and recording

a. Each animal is examined and observed for abnormalities at each monitoring point.
b. Each criterion is scored and the score marked on the recording sheet. Training is required to ensure all personnel are consistent in terms of scoring.
c. Scores are then added together and a **Total Welfare Impact Score** marked on the recording sheet.
d. Appropriate to the Total Welfare Impact Score, specific actions or interventions are undertaken.
e. Comments concerning abnormalities are recorded in the "Comments" section.
f. Any other abnormalities are recorded in the "Other" section.
g. Any abnormality that is observed to be of greater severity than the descriptions provided above will require immediate euthanasia of the animal.
h. All reporting documentation will be completed and submitted to facilities staff/manager and AWO if required.

© The University of Western Australia 2015

Monitoring recording sheet

1) ANIMAL DETAILS

AEC Protocol #	RA/3/100/1617	Weighing frequency	weekly	Strain	MHC403
Cage No.	–	Starting weight	36.8g	Age/DOB	9/11/2018
Animal No.	501	Weight with 10.% weight loss (Threshold 1)	—	Sex	male

2) MONITORING

**Day**	Mon						
**Date**	14/10/19						
**Time**	14:10	14:25	15:30	16:30			
**Procedure**	before ECHO, ECG	ISO Inj	1hr post	2hr Post ECHO, ECG			
**Criteria**							
Activity – i.e. movement around the cage<br>Bright, Alert, Responsive (BAR)	0	2*	2*	2*			
Body posture	0	0	0	0			
Social behaviour	–	–	–	–			
Coat quality/skin condition	0	0	0	0			
Body Condition	0	0	0	0			
Facial Expression	0	0	0	0			
Injection Site	–	0	0	0			
Other	–	–	–	–			
**Total**	0	2	2	2			
**Signature**	[illegible]	[illegible]	[illegible]	[illegible]			

Comments: 2* ⇒ animal is not walking up/still.

© The University of Western Australia 2013 CRICOS Provider Code: 00126G

Monitoring recording sheet

1) ANIMAL DETAILS

AEC Protocol #	RA/3/100/1617	Weighing frequency	weekly	Strain	MHC403
Cage No.	—	Starting weight	36.6g	Age/DOB	9/11/18
Animal No.	503	Weight with 10.% weight loss (Threshold 1)	—	Sex	male

2) MONITORING

**Day**	Mon						
**Date**	14/10/19						
**Time**	14:40	15:00	16:00	17:00			
**Procedure**	ECHO, ECG	130 inj.	1hr post	2hrs post ECHO, ECG			
**Criteria**							
Activity – i.e. movement around the cage<br>Bright, Alert, Responsive (BAR)	0	2*	2*	2			
Body posture	0	0	0	0			
Social behaviour	—	—	—	—			
Coat quality/skin condition	0	0	0	0			
Body Condition	0	0	0	0			
Facial Expression	0	0	0	0			
Injection Site	—	0	0	0			
Other	—	—	—	—			
**Total**	0	2	2	2			
**Signature**	[illegible]	[illegible]	[illegible]	[illegible]			

Comments: 2* ⇒ animal is not moving yet still

© The University of Western Australia 2013 CRICOS Provider Code: 00126G

Monitoring recording sheet

1) ANIMAL DETAILS

AEC Protocol #	RA/3/100/1617	Weighing frequency	weekly	Strain	MHC403
Cage No.	–	Starting weight	34.5g	Age/DOB	1/12/18
Animal No.	508	Weight with 10.% weight loss (Threshold 1)	–	Sex	male

2) MONITORING

**Day**	Mon						
**Date**	18/11/19						
**Time**	9:30	9:45	10:45	11:45			
**Procedure**	ECHO, ECG	ISO inj.	~~ECHO, ECG~~ 1hr post	ECHO, ECG 2hr post			
**Criteria**							
Activity – i.e. movement around the cage<br>Bright, Alert, Responsive (BAR)	0	2*	2	2			
Body posture	0	0	0	0			
Social behaviour	–	–	–	–			
Coat quality/skin condition	0	0	0	0			
Body Condition	0	0	0	0			
Facial Expression	0	0	0	0			
Injection Site	–	0	0	0			
Other	–	–	–	–			
**Total**	0	2	2	2			
**Signature**	[illegible]	[illegible]	[illegible]	[illegible]			

Comments: 2* → mouse is not moving around / still

© The University of Western Australia 2013 *CRICOS Provider Code: 00126G*

Monitoring recording sheet

1) ANIMAL DETAILS

AEC Protocol #	RA/3/100/1617	Weighing frequency	weekly	Strain	MHC403
Cage No.	—	Starting weight	34.7	Age/DOB	2/12/13
Animal No.	513	Weight with 10 % weight loss (Threshold 1)	—	Sex	male

2) MONITORING

**Day**	Fri						
**Date**	06/12/19						
**Time**	10:35	10:50	11:50	12:50			
**Procedure**	ECHO, ECG	180 mg	1 hr post	2 hr post ECHO, ECG			
**Criteria**							
Activity – i.e. movement around the cage<br>Bright, Alert, Responsive (BAR)	0	2*	2*	2*			
Body posture	0	0	0	0			
Social behaviour	—	—	—	—			
Coat quality/skin condition	0	0	0	0			
Body Condition	0	0	0	0			
Facial Expression	0	0	0	0			
Injection Site	—	0	0	0			
Other	—	—	—	—			
**Total**	0	2	2	2			
**Signature**	[signature]	[signature]	[signature]	[signature]			

Comments: 2* → mouse is not rolling up

© The University of Western Australia 2013 CRICOS Provider Code: 00126G

Monitoring recording sheet

1) ANIMAL DETAILS

AEC Protocol #	RA/3/100/1617	Weighing frequency	weekly	Strain	MHC403
Cage No.	—	Starting weight	39.3g	Age/DOB	21/12/13
Animal No.	514	Weight with 10.% weight loss (Threshold 1)	—	Sex	male

2) MONITORING

**Day**	Mon						
**Date**	9/12/13						
**Time**	10:40	11:00	12:00	13:00			
**Procedure**	ECHO, ecg	ISO ip	1hr post	2hr post echo, ECG			
**Criteria**							
Activity – i.e. movement around the cage<br>Bright, Alert, Responsive (BAR)	0	2*	2*	2			
Body posture	0						
Social behaviour	—	—	—	—			
Coat quality/skin condition	0	0	0	0			
Body Condition	0	0	0	0			
Facial Expression	0	0	0	0			
Injection Site	—	0	0	0			
Other	—	—	—	—			
**Total**	0	2	2	2			
**Signature**	[illegible]	[illegible]	[illegible]	[illegible]			

Comments: 2* → means that mouse is not waking up / still

© The University of Western Australia 2013
CRICOS Provider Code: 00126G

Monitoring recording sheet

1) ANIMAL DETAILS

AEC Protocol #	RA/3/100/1617	Weighing frequency	weekly	Strain	MHC403
Cage No.	—	Starting weight	35.9	Age/DOB	21/12/18
Animal No.	515	Weight with 10.% weight loss (Threshold 1)	—	Sex	male

2) MONITORING

**Day**	Mon						
**Date**	9/12/19						
**Time**	10:00	10:15					
**Procedure**	ECG, echo	Iso inj.					
**Criteria**							
Activity – i.e. movement around the cage<br>Bright, Alert, Responsive (BAR)	0						
Body posture	0						
Social behaviour	–						
Coat quality/skin condition	0						
Body Condition	0						
Facial Expression	0						
Injection Site	–						
Other	–						
**Total**	0						
**Signature**	[illegible]						

Comments:

↑ died after Iso inj, during recording

© The University of Western Australia 2013 *CRICOS Provider Code: 00126G*

Monitoring recording sheet

1) ANIMAL DETAILS

AEC Protocol #	RA/3/100/1617	Weighing frequency	weekly	Strain	MHC403
Cage No.	–	Starting weight	39.13	Age/DOB	21/12/18
Animal No.	517	Weight with 10.% weight loss (Threshold 1)	—	Sex	male

2) MONITORING

**Day**	Tue						
**Date**	3/12/19						
**Time**	11:10	11:30	12:30	13:30			
**Procedure**	echo, ECG	180	1hr 180	2hr 180 echo, ECG			
**Criteria**							
Activity – i.e. movement around the cage<br>Bright, Alert, Responsive (BAR)	0	2*	2*	2*			
Body posture	0	0	0	0			
Social behaviour	—	—	—	—			
Coat quality/skin condition	0	0	0	0			
Body Condition	0	0	0	0			
Facial Expression	0	0	0	0			
Injection Site	—	0	0	0			
Other	—	—	—	—			
**Total**	0	2	2	2			
**Signature**	[illegible]	[illegible]	[illegible]	[illegible]			

Comments: * – mouse is still, not awake

© The University of Western Australia 2013 CRICOS Provider Code: 00126G

Monitoring recording sheet

1) **ANIMAL DETAILS**

AEC Protocol #	RA/3/100/1617	Weighing frequency	weekly	Strain	MHC403
Cage No.	—	Starting weight	38.11	Age/DOB	6/4/19
Animal No.	570	Weight with 10 % weight loss (Threshold 1)	—	Sex	male

2) **MONITORING**

**Day**	Wed						
**Date**	18/03/20						
**Time**	13:50	14:10	15:10	16:10			
**Procedure**	Echo, ECG	op / 1⁰	1hr post 1⁰	2hr post 1⁰, ecg, echo			
**Criteria**							
Activity – i.e. movement around the cage<br>Bright, Alert, Responsive (BAR)	0	2*	2*	2			
Body posture		0	0	0			
Social behaviour	—	—	—	—			
Coat quality/skin condition		0	0	0			
Body Condition		0	0	0			
Facial Expression	0	0	0	0			
Injection Site	—	0	0	0			
Other	—	—	—	—			
**Total**	0	2	2	2			
**Signature**	[illegible]	[illegible]	[illegible]	[illegible]			

Comments: 2* – mouse is still, not moving, not rolling up, breathing normal.

© The University of Western Australia 2013
CRICOS Provider Code: 00126G

Monitoring recording sheet

1) ANIMAL DETAILS

AEC Protocol #	RA/3/100/1617	Weighing frequency	weekly	Strain	MHC403 α/+
Cage No.	—	Starting weight	34.5g	Age/DOB	1/12/18
Animal No.	503	Weight with 10.% weight loss (Threshold 1)	—	Sex	male

2) MONITORING

Day	Mon						
**Date**	18/11/19						
**Time**	10:00	10:20	11:20	12:20			
**Procedure**	Echo, ecg	180 inj: cho, ecg	1hr post	2 hrs post			
**Criteria**							
Activity – i.e. movement around the cage<br>Bright, Alert, Responsive (BAR)	0	1	0	0			
Body posture	0	0	0	0			
Social behaviour	–	–	–	–			
Coat quality/skin condition	0	0	0	0			
Body Condition	0	0	0	0			
Facial Expression	0	0	0	0			
Injection Site	–	0	0	0			
Other	–	–	–	–			
**Total**	0	[illegible]	0	0			
**Signature**	[illegible]	[illegible]	[illegible]	[illegible]			

Comments:

© The University of Western Australia 2013
CRICOS Provider Code: 00126G

Monitoring recording sheet

1) ANIMAL DETAILS

AEC Protocol #	RA/3/100/1617	Weighing frequency	weekly	Strain	MHC403 wt
Cage No.	—	Starting weight	30.2 g	Age/DOB	18/2/2019
Animal No.	536	Weight with 10.% weight loss (Threshold 1)	—	Sex	male

2) MONITORING

**Day**	The						
**Date**	17/12/19						
**Time**	9:20	9:45	10:45	11:45			
**Procedure**	echo/ecg	120 ip echo, ecg	1hr post	2 hr post			
**Criteria**							
Activity – i.e. movement around the cage<br>Bright, Alert, Responsive (BAR)	0	2	1	0			
Body posture	0	0	0	0			
Social behaviour	—	—	—	—			
Coat quality/skin condition	0	0	0	0			
Body Condition	0	0	0	0			
Facial Expression	0	0	0	0			
Injection Site	—	0	0	0			
Other	—	—	—	—			
**Total**	0	2	1	0			
**Signature**	[illegible]	[illegible]	[illegible]	[illegible]			

Comments:

© The University of Western Australia 2013 CRICOS Provider Code: 00126G

Monitoring recording sheet

1) ANIMAL DETAILS

AEC Protocol #	RA/3/100/1617	Weighing frequency	weekly	Strain	MHC403 wt.
Cage No.	–	Starting weight	33.0g	Age/DOB	23/2/19
Animal No.	548	Weight with 10.% weight loss (Threshold 1)	–	Sex	male

2) MONITORING

**Day**	Tue						
**Date**	17/12/19						
**Time**	10:00	10:20	11:20	12:20			
**Procedure**	[illegible]	120 [illegible]	1hr [illegible]	2hr [illegible]			
**Criteria**							
Activity – i.e. movement around the cage<br>Bright, Alert, Responsive (BAR)	0	1	0	0			
Body posture	0	0	0	0			
Social behaviour	–	–	–	–			
Coat quality/skin condition	0	0	0	0			
Body Condition	0	0	0	0			
Facial Expression	0	0	0	0			
Injection Site	–	0	0	0			
Other	–	–	–	–			
**Total**	0	[illegible]	0	0			
**Signature**	[illegible]	[illegible]	[illegible]	[illegible]			

Comments:

© The University of Western Australia 2013
CRICOS Provider Code: 00126G

Monitoring recording sheet

1) ANIMAL DETAILS

AEC Protocol #	RA/3/100/1617	Weighing frequency	weekly	Strain	MHC403 wt
Cage No.	–	Starting weight	34.8g	Age/DOB	2/4/19
Animal No.	559	Weight with 10.% weight loss (Threshold 1)	–	Sex	male

2) MONITORING

**Day**	Tue						
**Date**	17/12/19						
**Time**	12:50	13:20	14:20	15:20			
**Procedure**	echo, ep	1h, ip echo, ep.	1hr [illegible] post	2hrs post [illegible]			
**Criteria**							
Activity – i.e. movement around the cage<br>Bright, Alert, Responsive (BAR)	0	1	0	0			
Body posture	0	0	0	0			
Social behaviour	–	–	–	–			
Coat quality/skin condition	0	0	0	0			
Body Condition	0	0	0	0			
Facial Expression	0	0	0	0			
Injection Site	–	0	0	0			
Other	–	–	–	–			
**Total**	0	1	0	0			
**Signature**	[illegible]	[illegible]	[illegible]	[illegible]			

Comments:

© The University of Western Australia 2013
CRICOS Provider Code: 00126G

Monitoring recording sheet

1) ANIMAL DETAILS

AEC Protocol #	RA/3/100/1617	Weighing frequency	weekly	Strain	MHC403 wt
Cage No.	–	Starting weight	40.9	Age/DOB	6/4/19
Animal No.	565	Weight with 10 % weight loss (Threshold 1)	–	Sex	male

2) MONITORING

**Day**	Tue						
**Date**	17/12/19						
**Time**	13:30	14:05	15:00	16:00			
**Procedure**	echo ecg	180 ip					
**Criteria**							
Activity – i.e. movement around the cage<br>Bright, Alert, Responsive (BAR)	0	2	1	0			
Body posture	0	0	0	0			
Social behaviour	–	–	–	–			
Coat quality/skin condition	0	0	0	0			
Body Condition	0	0	0	0			
Facial Expression	0	0	0	0			
Injection Site	–	0	0	0			
Other	–	–	–	–			
**Total**	0	2	1	0			
**Signature**	[illegible]	[illegible]	[illegible]	[illegible]			

Comments:

© The University of Western Australia 2013
CRICOS Provider Code: 00126G

Monitoring recording sheet

1) ANIMAL DETAILS

AEC Protocol #	RA/3/100/1617	Weighing frequency	weekly	Strain	MHC403 wt
Cage No.	–	Starting weight	~~[illegible]~~ 37.5	Age/DOB	23/5/2019
Animal No.	581	Weight with 10.% weight loss (Threshold 1)	—	Sex	male

2) MONITORING

**Day**	Wed.						
**Date**	18/3/20						
**Time**	9:30	9:50	10:50	11:50			
**Procedure**	echo, iso	150, ip echo, iso	1 hr post 150	2 hr post 150			
**Criteria**							
Activity – i.e. movement around the cage<br>Bright, Alert, Responsive (BAR)	0	1	0	0			
Body posture	0	0	0	0			
Social behaviour	–	–	–	–			
Coat quality/skin condition	0	0	0	0			
Body Condition	0	0	0	0			
Facial Expression	0	0	0	0			
Injection Site	–	0	0	0			
Other	–	–	–	–			
**Total**	0	1	0	0			
**Signature**	[illegible]	[illegible]	[illegible]	[illegible]			

Comments:

© The University of Western Australia 2013
CRICOS Provider Code: 00126G

Monitoring recording sheet

1) ANIMAL DETAILS

AEC Protocol #	RA/3/100/1617	Weighing frequency	weekly	Strain	MHC403 wt.
Cage No.	—	Starting weight	38.56	Age/DOB	23/5/19
Animal No.	583	Weight with 10.% weight loss (Threshold 1)	—	Sex	male

2) MONITORING

**Day**	Wed						
**Date**	180320						
**Time**	10:00	10.25	11:30	12:30			
**Procedure**	echo, ecg	150 ip ecg, echo	1hr post ip	2hr post ip			
**Criteria**							
Activity – i.e. movement around the cage<br>Bright, Alert, Responsive (BAR)	0	2	0	0			
Body posture	0	0	0	0			
Social behaviour	—	—	—	—			
Coat quality/skin condition	0	0	0	0			
Body Condition	0	0	0	0			
Facial Expression	0	0	0	0			
Injection Site	—	0	0	0			
Other	—	—	—	—			
**Total**	0	2	0	0			
**Signature**	[illegible]	[illegible]	[illegible]	[illegible]			

Comments:

© The University of Western Australia 2013
CRICOS Provider Code: 00126G
